# An Essay on the Remittent and Intermittent Diseases Including Generically, Marsh Fever, and Neuralgia, &c. &c.

**Published:** 1830

**Authors:** 


### REVIEWS AND BIBLIOGRAPHICAL NOTICES.

---

ART.—VIII. *An Essay on the Remittent aud Intermittent Diseases, including Generically, Marsh Fever and Neuralgia, &c. &c.* By JOHN M'CULLOUGH, M. D., &c.

[*Concluded from the last Number.*]

We resume the analysis of Dr. M'Cullough's complex and extended work. Such of our readers as may have held out to the end of what we placed before them in our last number, will recollect that we broke off in the midst of the consequences of intermittent fever ; having in our closing paragraph presented the author's views of the influence of that disease, in destroying or injuring the intellectual faculties. Intimately connected with these melancholy effects, are those which this malady exerts upon the general sensibilities of the system. In illustration of which he has the following remarks :

"That this disease produces an insensibility to pleasurable impressions, if I have but recently mentioned it, is a fact too remarkable in its results not to be noticed here as a part of the general illustration of this condition of the nervous system. Nor is that consequence a secondary one, originating in false or perverted moral views, or in an aberration of the reasoning faculties ; since it appears, on the contrary, where that does not exist, or to be, absolutely, an insensibility, or a primary disorder, in the nerves of those organs of sense, which are the mediums of pleasure. I cannot doubt this, from the facts related to me by patients thus suffering, and who were at the same time good metaphysicians ; those facts and their complaints being, that beautiful objects, such as pictures, natural scenery, and so forth, which, before that, or when in health, had been most pleasurable or engaging, seemed to make no impression at all, on the sense ; and not because their minds were affected, because these things were viewed under a state of mental anxiety or derangement, since that state of mind was not present, but simply from failing to make the usual and expected impressions. From such patients I have received also the same complaints and statements, with respect to the other usual causes of simple pleasurable feelings ; and very particularly from those who, as musicians, were accustomed to delight in music, not less from science than feeling ; those being, that they seemed to suffer under a positive insensibility as to what used to be a source of the most refined delight, although labouring under no affection of the temper, nor any of those sensations commonly called hypochondriacal. And thus have others complained that the most grateful odours had ceased to give pleasure, that the scent of a rose was not only powerless, but produced absolute pain by reminding them of what it once was ; while every attempt to revive the former associations connected with this and other similar objects of delight, was unavailing." 159—160.

In this short history of a perverted state of all the animal sensibilities, resulting from *Malaria*, the reader will observe a remarkable coincidence with the morbid condition of the same sensibilities, the consequence of a severe *burn*, as reported in the last number of our journal.

Our author attributes much of the mischief just described, to the injudicious use of blood letting. In support of this opinion, he relates two cases, which fell under his own observation. We shall transcribe one of them.

"The first of these was a patient who had been subject to repeated attacks of quotidian, in successive years, under my own care, while a vigorous and intelligent young man of twenty-five, in the upper ranks of life. In a subsequent and similar attack during my absence, it was the fancy of his physician to prescribe blood-letting within the first days of the disease, under some erroneous view of its nature. No very obvious consequence followed the first operation ; but on the second, it was remarked that he ceased to complain as he had done, which appeared to give the temptation to a third trial ; immediately after which he fell into a state little short of idiotism, while the quotidian became also attended with many anomalous symptoms. Thus I found him, and just in time to prevent a fourth bleeding, which would probably have produced an extension of these consequences, and might easily have terminated, as I have more than once seen, in palsy, epilepsy, or death. The disease was cured in the usual way, by bark ; but the debility of mind was not removed for six months, while I have reason to suspect that it was even much more durable." 161.

On the subject of Malaria, as a simulating form of chronic intermittent, Dr. M. observes—

"I have seen four cases at least of absolute and pure mania, of most distinct tertian and quotidian forms, which, from this ignorance, as it appeared to me, became the subjects of gross mistreatment. The exacerbations were, in all, as distinct as those of a common intermittent would have been, and as regular, while the two tertian cases left a day intermediate of the most entire sanity of mind : the period of the paroxysm being a perfect mania, and not a febrile delirium, and while, in fact, no marks of fever could be discovered. And in one of these at least, the cause was as easily as it was distinctly traced ; since the disorder had commenced as a common tertian, which seemed to have entirely disappeared to be exchanged for the mania : while of the other, whatever my suspicions were, I could not procure a distinct previous history, from the inattention and ignorance of the friends and the patient. In the quotidians, not only were the accessory, if obscure, symptoms of intermittent to be found on an accurate inquiry, in both the cases, but while the original cause, in one of them, was distinctly traced, so was the truth of the conclusion confirmed by the success of the method of cure which was adopted, after great maltreatment." 163.

Passing, in defiance of a natural method, from these affections of the brain, to those of the stomach, our author speaks of vomiting, as an attendant on the paroxysms of an intermittent; and informs his readers, that he has seen this symptom return at stated periods for three months in the same patient. We have ourselves met with a distinctly marked quotidian cholera, while it is familiar to the profession, that the paroxysms of an intermittent are generally accompanied with nausea and other gastric affections, leaving the patient, in the intermission, so free from any disorder of the stomach, that his appetite is keen and his digestion good. All this is what a Broussaist finds little difficulty in comprehending ; believing, as he does, that intermittents are radicated in the stomach. In the opinion of Dr. M. such cases furnish us with examples of a 'real misdirection of the febrile paroxysm' or concentration of its energy upon a particular organ. In the ordinary means employed by the profession for the relief of this symptom, as it is called, Dr. M.

has no confidence, and would rely on nothing that is not, in its nature, qualified to cure the entire disease.

As might be expected, in the general derangements of the nervous function produced by intermittents, hysteria occasionally shows itself. Dr. M. has seen the *globus hystericus* recur for months, at daily regular periods, and known it cured, at last, by the remedies for chronic intermittent. We have repeatedly seen hysterical affections complicated with fever from Malaria, and can readily conceive, that in persons prone to nervous disorders, they might become the prominent symptoms, and divert the attention of the medical attendant from the real malady. Complications of this kind we have generally found unmanageable, because the condition of the nervous system on which they depend, is unfavourable to the restoration of health.

Closely allied to these affections, are palpitations of the heart, to which our author has devoted several pages. In his opinion they are true neuralgias of the organ, and unaccompanied with pain, only because of the absence of nerves of sensation—the heart being endowed with scarcely any other than nerves of motion. Under this view, the term neuralgia or nerve *ache*, is not very appropriate ; but we may understand it to mean, a morbid state of the cardiac nerves, analagous to that which constitutes the neuralgia or tic douloureux, of parts endowed with animal sensibility. When we call to mind how constantly and deeply the heart is implicated in all febrile affections, we cannot be surprized, to find our author insisting, that this organ experiences a great variety of irregular actions and palpitations in the obscure, relapsing, and chronic varieties of intermittent. That his views of this difficult and interesting subject may be fully understood, we shall present our readers with an extended extract.

"If I am, myself, inclined to consider the palpitation which is one of the most remarkable and the most distressing of the anomalous symptoms or forms of chronic intermittent, as a Neuralgia of the heart, as one of those local misdirections of this disease which, affecting other nervous organs or separate nerves, produces those very marked disorders, and if therefore my own desire would have been to have ranked it with those, and thus to have deferred it at present, I

am fearful of giving more cause, by these new views, for that incredulity and those objections which I foresee. I shall therefore treat of it here as a merely anomalous symptom of chronic intermittent; reserving to myself the privilege of a different and better classification, when the alarm at these novelties shall have subsided.

The modes, as to the patient's feeling and as to the recurrences, in which the heart is affected in these cases, are so various, that I must examine them at some length: a proceeding which becomes absolutely necessary, from the almost universal errors on this subject, from the pertinacity with which these cases are maintained to be independent nervous affections, or are referred to organic derangements that have no existence, and from the consequent maltreatment of the patients; to which are too often added alarm and anticipations, materially aggravating a disease which is in itself sufficiently distressing.

Increase of the velocity or of the strength of the circulation, or of both, attended by an increase in the force of the blow given by the apex of the heart, is the most simple of these affections, while this may be merely sensible to the patient himself, or else may be visible to a bystander. In other cases, that increase of action is attended by irregularities in the force; or there are occasional palpitations, of short duration, but repeated; or they are sometimes limited to single starts or spasms, or else there are intermissions under every irregularity that can be imagined. These disordered actions, which must be felt to be understood, are often attended with the most distressing sensations in the brain and in the whole system, affecting even the mind in various ways; producing among other things uncontrollable fear or inexplicable alarm, and sometimes seeming to threaten immediate death. When of any duration, they also often leave the patient exhausted, as if from the excess of fatigue; and this, constituting an inexplicable derangement, often lasts during a whole day, or long after the palpitation has ceased. In the heart itself, among other strange sensations, it is not uncommon to feel as if it was grasped by a hand, and compressed; while there is sometimes also the sensation of its being paralyzed, or about to cease from motion altogether. In other instances, and in the mildest cases, the affection is no greater than that which commonly attends fear, or that peculiar and slight alarm which may occur, for example, in a timid or anxious speaker about to rise in public; and, apparently in consequence of association, it is the effect of this to produce alarm, or fear, or anxiety, for which the patient, aware of its real cause, can find no reason, but which another, ignorant of this, or morbidly active in seeking for moral evils, easily refers to events or circumstances in life that are dreaded or anticipated. Or, as will be very obvious, the principle of association produces, by inversion, the passion or feeling which, in the ordinary cases, excite these peculiar actions of the heart. Hence it is, that to be aware of the nature of this disorder, is important to the patient; as, by explaining to him the true cause, it will prevent him from seeking for imaginary evils that will aggravate his sufferings.

Such are the more ordinary forms of this palpitation; and how often it has been referred to enlargement of the heart, to ossifications, and so on, I have seen in but too many instances. But I must also remark, that this diseased action sometimes takes place in the inferior aorta, as well as in the heart, or is occasionally transferred from this organ to the artery; constituting an affection well known to occur in what is called dyspepsia, which, for aught I know, may depend on many nervous diseases, but which, as before observed, I have often most clearly traced to the class of disorders under review. I need not remind the medical reader of the description of this particular disorder given by Dr. Baillie; nor ought I to pretend to conjecture the cause or causes as it did occur to him; but while I have traced it to the cause under review, I am bound to add that this inference was drawn from observing that it alternated in relapses, and indeed in periods of long duration, with a palpitation of the heart dependent on intermittent, and itself alternating with relapses of common quotidian, as well as with the intermittent periods themselves, in a case of long standing subject to many other anomalies.

If the real cause of these affections of the heart be often obscure, there ought never to be any difficulty in ascertaining their nature when any other symptoms of chronic intermittent are present, when the patient is constitutionally subject to this disease, or is known to have suffered formerly from it, or when, as frequently happens, this affection alternates in any manner, with any of the ordinary, or of the anomalous symptoms of intermittent. These form the easier grounds of judgment to the physician, often as they are all overlooked; and in these several modes do those derangements of the heart often occur.

Presuming that the disease is regular in its returns, and connected with obvious intermittent, the palpitation, as far as I have observed, belongs to the same period of the paroxysm as the cold fit, or generally so; while in cases where it is the only symptom, or the only very visible disorder, it has equally appeared to me to be the substitute for this. Thus also, when slight, it may usher in the paroxysm, subsiding in a short time; and here it corresponds with that general and common affection of the circulation which attends the commencement of the diurnal fit. The duration of a paroxysm of this kind, like the violence, is consequently variable; extending from an hour or less to twelve or more; possibly, in such cases, occupying the entire time of what would be a paroxysm in the common intermittent: and I have met with one instance of a double quotidian form, in which the two returns left very little repose to this organ during the twenty-four hours, and in which the patient was nearly deprived of all sleep, procuring it only during the very short intervals. This case, after lasting three months, while sentence of condemnation had also been passed against the patient, under the supposition of a disorganized heart, was cured in two days by the remedies of intermittent; proving clearly that the judgment which I had formed respecting it was correct." 166—169.

Palpitations (neuralgias) of the heart are generally in the opinion of Dr. M. connected with chronic intermittents, and in most cases, if not all, associated with some of their collateral symptoms ; which, if physicians had been aware of the connexion, would have been very commonly noted. Many obscurities and irregularities contribute, however, to confuse the judgment and mislead the practitioner.

"It happens that the fits of palpitation are interrupted, as I have just remarked ; and the analogy, in this case, will be found in those intermittents where the common paroxysm is similarly irregular, or where there is an intermixture of numerous cold and hot stages in one paroxysm. If there are two fits in a day, it may be a double quotidian form, such as I have just described ; while it is plain, that if regulated by the other complicated types of intermittent, there is scarcely an irregularity which it may not exhibit. If it is irregular in ways not reconcilable to those cases, its analogy will still be equally found in those chronic intermittents which consist in irregular returns, of an endless variety ; while, if only occasionally produced, and by some cause inducing debility, irritation, or perhaps mental affections, we have precisely the same appearances in the common chronic intermittent. And thus, in fact, do the fits of palpitation frequently occur, in patients who have been infested with chronic intermittents ; single attacks, lasting perhaps for a few hours, not to recur for some weeks or months, and often traceable to a marked cause.

One other mode and cause of irregularity must however be pointed out ; because, while it is needful to be known, it belongs to the proofs of the nature and cause of this disease. In this case, when the palpitation disappears, it is to be replaced by some other local symptom or anomaly, or else by a perfect intermittent, or by some irregular and partial form of this. In practice, here, the error is to suppose that the palpitation is a disease of itself, distinct from the others, be those what they may ; that the patient, for example, has a disorganized heart ; and an intermittent also, or some other disorder, or that the whole are symptoms of some nervous disease. A more careful observer, or one at least who is acquainted with this subject, will however, perceive that if the palpitation yields to an intermittent, it is because the disorder has changed its character ; or that if vomiting, or hysteria, or diarrhea, or any other of the symptoms so often mentioned, appear when this retires, it is because the local action of the chronic intermittent has taken a new direction. And thus, by watching, does he learn to explain what appeared to him under a false light, and to regulate his practice accordingly." 170—171.

It deserves to be known and recollected, that, while the palpitations are essentially the offspring of the fever, or of the Malaria which occasioned it, they are sometimes so much influenced by external or adventitious causes, such as position, exercise, &c. as to favour and perpetuate the idea, that they are dependent or organic læsions, when in reality they are parts of the fever, or substitutes for it.

"Thus, while a fit of palpitation can sometimes be removed by various means, so is it irritated, or augmented, or prolonged ; while these facts tend to mislead the practitioner as to the imagined cause and nature of the disorder, as they may also afford arguments to those who are unwilling to admit the explanation which I have here given. I may state the most extraordinary case of this nature which ever occurred to me ; because while it had been determined that organic and incurable disorder was present, it vanished suddenly on the appearance of an intermittent quotidian, which was soon afterwards cured.

In this case, the patient had no relief from the palpitation, which was both incessant and violent, except in the erect posture, when awake, while it returned instantly on sitting down, and while, frequently also, it could be removed only by walking about the room, in which manner he was compelled to pass nearly the whole day. Thus also, at night, was it instantly produced by attempting to lie down ; so that the little sleep that was procured, and not till after urgent necessity, was to be obtained only by being bolstered up as erectly as possible." 171.

Dr. M. believes in the identity of these palpitations, with many examples of what are called rheumatism of the heart, and, in evidence of his opinion, cites a case which we think it worth while to extract.

"In this patient, the palpitation was of the most severe character, and had lasted for some months, occupying a large part of every day, or recurring in successive fits, but without any great regularity, and always increased by irritations of various kinds. It was of that kind and character which seemed to justify the belief in an organic disease, as far as this ever can be conjectured : while there are no marks of temporary fever, or of any other derangement, to be traced, and while the patient was even robust, and, when not suffering extremely from the fits, capable of all the usual occupations of a very active life. After much cross-examination it was discovered that he had for many previous years been subject, annually, and nearly always at the same periods, commencing with the end of summer, to different diseases ; being in one season, periodical toothach, in another diarrheas, in a third rheumatism, and in others, anomalous affections of the urinary organs, dyspepsia, hypochondriasm, pulsations of the aorta, and further, to a marked, though a very slight, quotidian intermittent. Thus was my judgment of the true nature of the disorder formed, while it was confirmed by the further progress ; as it afterwards disappeared almost suddenly, to be replaced by a periodical rheumatism of the deltoid muscle, which was succeeded by "tic douloureux" or Neuralgia of the face, and, in succession, by quotidian intermittent and other Neuralgias ; that patient having in himself exhibited a perfect nosology of all the anomalies of chronic intermittent, of which this very marked and independent palpitation was one. And if this is one of the most perfect cases which I have met with to prove the cause of sueh palpitations, so does it afford a marked evidence, to be confirmed hereafter by much more, of the nature of all the Neuralgias, of their common connexion, and of their dependence on intermittent or their origin in Malaria." 173.

From palpitations our author proceeds to speak of palsy of the heart, of which he cites a curious case that fell under his own observation, and was connected with a chronic quotidian, but we have not room to notice it any further.

Having disposed of neuralgias of the heart, embracing what is vulgarly denominated rheumatism of that organ, our author, after an apology for his want of method, arrives at the study of rheumatism of the muscles and membranes, generally, which in his view is often, at once, a mode of neuralgia and of intermittent.

"The most simple case of all, while it is one that ought never to be mistaken, is that where a rheumatic pain in some particular muscle is strictly periodical, returning and ceasing in regular paroxysms. In such cases, the part affected may sometimes be exceedingly limited, occupying only a few fibres of a muscle, though, even then, the pain is often severe ; while in others, the extent may be very considerable. Thus even the whole body may suffer under it ; or rather there may be so many different muscles affected, in some place or other, that scarcely any movement can be made in which some one or more of the disordered portions is not brought into action ; conveying thus, to the patient, the feelings as of a universal rheumatism.

If such a periodical recurrence is not sufficient to satisfy a practitioner respecting the true nature of such a disease, it will often be found attended with other symptoms explanatory of its cause, while these also form the proofs of the propriety of thus considering and arranging it. Thus it will be found to occur in persons who have been, at other times, affected by intermittent: forming, in itself, a period of relapse, and a substitute for the more common modes of the chronic disease. In other cases, the rheumatic pains will alternate with any of the other marked symptoms belonging to this disease; as, in the case last described at so much length, it does with palpitation of the heart. Or it may cease on the appearance of the common symptoms of intermittent, or the patient may recollect that it had formerly existed and suddenly disappeared; without apparent cause, or from change of place, or from the action of feeble or fantastic remedies. And hence, I may remark by the way, it is, that so many absurd or imaginary remedies have gained credit in rheumatism generally; as it is the character of intermittents thus to cease of their own accord, or to yield to trifling changes, or even, as is abundantly notorious, to give way to remedies acting on the imagination; even to charms." 179.

To detect the true character of many cases of this kind, Dr. M. thinks is rendered difficult, by the prevailing prejudice, that rheumatism is always an original and distinct disease. Under this erroneous impression, many cases, which are really modes of obsure and chronic intermittent, are disposed of by the routine practitioner, under the convenient designation of 'chronic rheumatism.'

"It is plain that, under those ordinary and current views, the cases to which I have here been referring will generally fall under the division of chronic rheumatism, as the symptoms in question are the produce of a chronic disease. I do not pretend to enter into all the possible causes of chronic rheumatism, nor to conjecture what proportion of the cases which occur, be they local or more general, may be original diseases of a distinct nature, and what comparative number may belong to the cause under review. But it is probable that the latter cases do form a very large proportion of the chronic rheumatisms occurring, and that we are thus enabled to explain their inveteracy of duration, as well as their tendency to recur; properties of all the disorders arising from this cause: while it will also explain the action of the modes of cure that are successful in this disease. Hereafter, possibly, physicians may be induced to examine this disorder with more care, and by this light; and we may then perhaps approach somewhat nearer to a true view of a disease, of which our ignorance has hitherto been so disgraceful." 181.

From chronic, Dr. M. proceeds to acute rheumatism, which he presents to the consideration of his readers in the following manner—

"The question at present is, whether there are not acute rheumatisms of the most regular form, which are truly modes of the quotidian intermittent, or of the remittent, possibly, originating in the same causes : and if it shall be decided that this is the fact, and that there is also an acute rheumatism generically different, then we shall probably be able to explain the causes of the contests so long maintained respecting the use of bark in this disease.

The facts which would seem to prove this opinion, are chiefly these. There is a periodical exacerbation, if there is not always an absolute remission of the pains ; and the duration of the disease is very analogous to that of a remittent, or of one period of an intermittent. The causes correspond, if they are not identical, while the remedy is often the same ; since, after all that has been disputed, there is no doubt that many cases are cured by bark, and that blood-letting is not only often ineffectual, but pernicious ; its action altogether, being, in fact, very similar to that which it exerts on remitting and severe intermitting fevers.

Thus while, in acute rheumatism, the misapplication or abuse of blood-letting often produces the chronic disease, so does a similar practice frequently induce the chronic state of intermittent, or convert an acute and terminable case into a durable one. It is not impossible also that the termination of the pains of acute rheumatism, succeeded by affection of the brain, and so often producing death, may be an analogy to what happens in other cases of intermittent diseases, where one local affection is exchanged for another, or disappears to be replaced by an augmentation of the general fever : while I can further easily understand, that the misapplication or the injudicious excess of blood-letting in such a case, may absolutely produce that comatose state which sometimes occurs in this disorder, and which is so generally considered a transference of the inflammation ; since this is the very apoplectic state, if I mistake not, which is produced in the same manner in remittent and intermittent fevers. With what propriety therefore blood-letting is resorted to in the cases of this nature, will be abundantly palpable, should this view be but even sometimes correct." 181—182.

Devoting a page or two to rheumatic affections of the intercostal muscles, of the loins (lumbago,) and of the face, the first of which is often confounded with pleurisy, our author dismisses this branch of his subject, and passes to 'other diseases of a local or peculiar nature, connected with intermittent, as symptoms or modifications.'

Diarrhœa is one of these varieties. In its chronic form our author thinks he can discover much, which indicates its dependence on, or connexion with, obscure intermittents. He has seen it assume both a quotidian and tertian type. He has, also, met with a cough, dry, spasmadic, and recurring in paroxysms, in one case daily, which he feels bound to regard as bearing the same relation to intermittents.

We may observe, that in many cases of these maladies or symptoms, their proximate cause may be a diseased liver, the offspring of intermittent fever, as diarrhœa and cough are both known to result from hepatic derangements occasioned by other causes than Malaria. Dr. M. has, however, met with a catarrh, which makes quotidian attacks in summer, and has received the appellation of 'hay fever,' which he is inclined to regard as a variety of intermittent. We have ourselves, in several instances, known intermittent and remittent fevers, to make their onset with catarrhal symptoms, which could not be traced to any exposure to vicissitudes of weather.

When intermittent fever, or its cause, awakens disease of the lungs, the malady may be mistaken for phthisis. Dr. M. has cited several cases of this kind, which were speedily cured by the bark. We recollect many years ago, to have seen a case of true hectic, so much aggravated by the residence of the patient in a miasmatic atmosphere, that for a while the paroxysms took on the character of a true ague and fever, or at least the chill was, for a time, augmented to a shake; and a physician not previously acquainted with the case, would have pronounced it an ague complicated with pulmonary derangement. The patient died with the usual symptoms of consumption.

The last variety of the local anomalies of intermittent fever which our author mentions, is strangury, of which he has seen three well marked cases. In all, its nature was at first mistaken. They were cured by changing the treatment, and one of them especially, was removed in a very pointed manner by a few doses of quina.

In the conclusion of this long, and in many respects, interesting chapter, our author inquires how far he has been anticipated in his views by previous writers, but we shall pass over the results of this investigation to enter upon the cure of intermittents.

This makes the subject of Chapter VII. which runs through nearly 40 pages, but we do not propose to dwell very long upon them. Dr. M. begins, indeed, with the candid declaration, that he has nothing strictly new to present, under this head. The aim of his book is not to set forth the treatment of marsh fever, but its associations and consequences, many of which he thinks have not hitherto been understood or even suspected to exist, by the profession.

Of the method of cure which he prefers, we cannot say has invented, a brief summary must suffice.

Declining to enter upon the treatment of the severe and malignant intermittents of hot climates, his *methodus medendi* is adapted to the simple intermittents of the north. He first speaks of charms, amulets, spiders, abracadabras, and other contrivances for making a strong impression on the senses, the feelings, and the imaginations of the sick; by which, not only intermittent fever, but many other diseases are sometimes cured.

His next class of remedies comprises alcohol, laudanum, cayenne and other diffusible stimulants, which administered before the chill are frequently found to avert the paroxysm. Of the whole, we regard opium and its preparations, at once the most efficacious, and the most harmless when unsuccessful. We are not prepared to believe, that it is entirely in virtue of a simple, exciting power, that the medicines of this class ward off the fits of an intermittent; for, if we are not greatly mistaken, the union of tartar emetic, in a large dose, with opium, increases the efficacy of the latter as a preventive of the paroxysm; while no one can pretend that the stimulating effect is not, thereby, diminished rather than increased. Of administrations in anticipation of the chill, we entertain from experience a favourable opinion; but it must be confessed, that they sometimes augment the hot fit, and, especially, that they may increase the cerebral affection. They have seemed to us particularly beneficial, when they excited the functions of the skin. Hence much of the benefit from an emetic, from the union of sudorifies with narcotics, from lying in bed and drinking warm herb teas, and from the external application of heat. If any great function, such as the cutaneous, *can* be kept up by our efforts to ward off the paroxysm, they will be successful.

For moderating the cold stage, he advises hot drinks, and external warmth, and prohibits bleeding and purging; both of which, however, when it is protracted to an unusual duration in strong constitutions, have been found useful. Indeed, we have repeatedly met with cases, in which the pulse, *before and throughout* the cold stage, was strongly excited, the patient at the same time suffering with thirst, head ache, back ache, pain in his joints, and sometimes delirium. In such cases, to throw in diffusible stimulants during the chill, can work out no other effect than to aggravate the hot stage. Of emetics in the former, he entertains a better opinion, in which we concur ; but if the constitution of the patient be shattered, they should be combined with laudanum—a union productive of the most agreeable effects, in all cases of great debility or nervous irritation, where emetic medicines are indicated.

As to the hot stage, while, from the very constitution of the disease it will subside if left to itself, it cannot be denied, that the patient, thus abandoned, is often left by it much worse than it found him ; and, therefore, it is our duty as far as possible, to mitigate its violence and limit its duration. Moreover, it is during this stage, that we may prepare the patient for the reception of the bark or its preparations. We are aware, that there are physicians who regard a preparation of the system for this purpose, as a work of supererogation, but as a general rule, we can by no means agree with them. Two modes of preparation, according to our own experience and reflections, are important. First—If the patient be of a sanguine temperament and plethoric habit, the quantity of blood and the power of the heart and vessels, should be reduced. Secondly—As the stomach and bowels are very commonly over charged with unhealthy secretions from the liver, and mucous membrane, they should be evacuated. If the former be not attended to, bark and diffusible stimulants may awaken inflammation ; while, until the latter is corrected, those medicines are often rejected, or if retained, cannot produce their specific effects.

In addition to blood letting, or in most cases without it, emetico-cathartics are important in the hot stage, as contributing to fulfil the indications just named, not less than to alleviate the sufferings of the patient and shorten their continuance. Of the formulæ for these objects, there are, among a multitude, two from which we have derived so much aid, in a long course of practice, that we should be ungrateful to the eminent men to whom we were indebted for them, not to make an acknowledgment of our obligation. The first is the union of tartarized antimony with the sulphate of soda or magnesia, both in *dilute* solution, and administered in 'broken doses,' till free evacuations take place. Our knowledge of this little recipe was derived, in our pupilage, from Dr. Robert Jackson's Treatise on the Fevers of Jamaica, and the Intermitting Fever of the United States, republished in Philadelphia, 35 years ago, and, in our humble opinion, one of the most valuable and unpretending practical works, which has appeared either before or since. The second formula, is from our distinguished, but strangely neglected countryman, the late professor Rush, whose writings, in less than 20 years from the time when he exercised an almost absolute sway over the medical mind of the United States, have gone out of fashion, and are read by no one. It consists of the well known union of tartarized antimony, calomel and the nitrate of potash. Our readers will observe, that both formulæ contain a refrigerant, neutral salt, on which, we are constrained to believe, not a little of their antiphogistic power depends ; and which gives to them, in the case, under review, so decided a superiority over the irritating and drastic cathartics.

In the cure of intermittents, Dr. M. lays but little stress on any thing, except bark and arsenic, and their preparations ; a sentiment in which we entirely concur. We have seldom seen agues (vernal excepted, which will often 'get well of themselves') cured without the administration of one or other of those medicines, or of their chemical preparations. Disputes have, now and then been gotten up, as to the administration of the bark in the early stages of intermittent. It is not our intention to enter the list, but we may observe, *en passant*, that if the active course of depletion which we have advocated, has been employed in the first paroxysms, the bark may be given, with safety and effect, at a much earlier period than it otherwise could ; and thus the aggregate of the patients sufferings be greatly diminished. Moreover, in malignant intermittents, it cannot be doubted that the exhibition of the bark immediately after an emetic, conjoined as it should be with laudanum, or without any previous preparation, may not only be safe, but proper and necessary. As to the quantity in which it should be given, an equal diversity of opinion exists, and the amount has varied from a drachm to an ounce, at a single dose !. This has partly arisen from difference of character in physicians, and partly from difference of character in epidemics. On the whole, we incline to liberal doses, as most effective in arresting the disease ; and concur in the practice of those physicians of the Western country and elsewhere, who have met certain epidemics with immense doses of this, their only known antidote. Different *corrigenda* have been united with the bark, a practice which has, by some, been condemned. It may be admitted that they do not augment its inherent curative powers, but they, sometimes, answer other valuable ends. Thus cayenne, cinnamon, or cloves, may reconcile the stomach to its first impress, and thereby promote the retention of the requisite quantity ; jalap, as proposed long ago by Senac, will maintain the peristaltic action of the bowels, which should never be allowed to flag ; laudanum will restrain the same action when too great ; the serpentaria will keep up the functions of the skin ; and the super tartrate of potash sustain that of the kidneys ; all of which are aids to the specific influence of our medicine, in arresting the recurrence of the paroxysms, which all of them, unaided by *it*, could not in general accomplish.

Of the relative powers of the bark and the preparations of quina, we shall say nothing. In general they both succeed, and sometimes both fail. We consider the bark the more stimulating of the two, and, therefore, better adapted to pretracted cases. The best mode of administering the quina, we believe to be in large occasional doses, in substance, and in powder instead of pills. United with opium or Dover's powder, and given before the chill, its effects are often admirable. When it produces constipation, aloes, in subtle powder, is an excellent corrective. They should be intimately blended and formed into a linctus or bolus.

Arsenic, with Dr. M. is, on the whole, a favourite medicine, and constitutes as we shall hereafter see, one of his two principal remedies for the distressing neuralgias, which he thinks of marsh origin ; and which are presently to occupy our attention. He prefers to give it in substance, and recommends a sixteenth of a grain, rubbed down with loaf sugar, 3 or 4 times a day. The late eminent professor Barton, in his lectures, recommended the following formula, which we prefer to any other.—

R. Acidi Arsenici gr. j.
Pulv. Opii. gr. iv.
Misce fiat pil. xvj.

Dr. M. thinks, that in proportion as an intermittent, in its pathological characters, approaches more nearly to a remittent, arsenic sinks below the bark in efficacy ; and that, moreover, its value is very small in protracted cases. On these points however, we would remark, that when the fever approaches to a remittent type, the bark itself is in most cases improper ; and that in chronic intermittents arsenic, *is* efficacious, if it be united with opium, and the patient at the same time be duly stimulated. A recent and simple intermittent, particularly of a tertian type, is that, in which Dr. M. has found it most effective.

Our author is not, of course, insensible to the great importance of change of place, in chronic intermittents. While the patient continues to inhale or *swallow* Malaria, it is not always possible, by the art of medication, to make him a sound man. In every protracted and obstinate intermittent, the subject of it should, if possible, be removed to a healthier situation, where the disease has often ceased without medicines, or yielded to their power which before was inoperative.

Dr. M. has devoted several pages to the diet and drinks of persons inhabiting aguish situations, or affected with the disease in its chronic form. If his motto is not quite, *vive la gourmandise*, he evidently looks with an eye of toleration upon the *bon vivans*. He thinks, that a much greater number are injured by a defective, than an excessive diet, and cites many facts in support of his proposition. On the fertile banks of the Ohio, abounding in corn and whiskey, if not in 'corn and wine,' we have too few examples of habitual starvation, to render us competent judges ; but on the whole we are inclined to subscribe to his doctrine ; and to adopt his opinion, that the animal body is better prepared to dispose of a superfluous diet, than to supply the deficiences of that which is too sparing and innutricious.

With these remarks we shall dismiss the subject of fever, in its more palpable type, and proceed to the analysis of the second and more interesting part of his work, which treats of the various NEURALGIÆ.

In the common parlance of the profession, there are '*nervous*' and '*spasmodic*' pains, which every student is taught to consider as widely different from '*inflammatory*' pains. Loose and exceptionable as these phrases are, it is undeniable that they are intended to indicate very different pathological states. Of the latter we are supplied with examples, in the *furunculus* or common boil, and in acute pleurisy ; while as familiar specimens of the former, we may cite flatulent colic, certain varieties of tooth ache, sick head ache, and the painful suborbitar affection of the face, denominated *tic douloureux*. In these two groups of maladies, the nerves of course are affected, otherwise there could be no pain ; but in one case the affection is connected with a manifest inflammatory plethora of the part ; while in the other no such engorgement in general exists, and they even appear, in some cases, accompanied by an opposite condition of the circulation. Now it is to this class of pains (nervous or spamodic) in the popular vocabulary of physic, that the elegant and classical cognomen NEURALGIA has recently been applied. At first it was, perhaps, intended as a mere substitute for the vulgar French epithet, *tic douloureux* ; but has at length, by Dr. M. and other writers, been extended to a great number of affections which are supposed to depend on the same pathological state, with that painful disorder. The tabular view prefixed to this article, has, already, given the reader an idea of the number and variety of the affections, which our author has marshaled under this new head ; and on this arrangement rests one of his claims to the merit of being a discoverer ; the other is associated with it, and consists in the enunciation, that these various neuralgiæ, are in general, the offspring, direct or indirect, of Malaria,—direct when produced without a remitting or intermitting fever—indirect when preceded by that disease. To the establishment of these two general propositions, and the developement of the corollaries, which depend on them, Dr. M. has, as he informs us, devoted many years of observation and reflection ; the fruits of which are spread before the profession, in a series of chapters, which make up more than half his book.

The neuralgia, vulgarly denominated tic douloureux, first receives attention, as being what naturalist call the prototype of the genus. After protesting against limiting the term to a painful affection of the suborbitar branch of the fifth pair of nerves, our author proceeds to describe a paroxysm of this deplorable malady, in which he endeavours to show its resemblance to a paroxysm of intermittent fever ; and as his manner of viewing this subject, is in some degree applicable to all the other affections which he refers to the same class, we shall make a liberal extract.

"Immediately before the attack, if the pulse is examined, it will be found to put on that character which it possesses in the cold stage of an intermittent ; while, through the progress of the poroxysm, it passes through the other analogous changes. If also a watchful patient, at least when directed to do so by his physician, (which I fear has rarely been the case,) attends to his previous feelings, he will find that there are most commonly some indications of a cold stage, generally obscure, it is true, as is the case in most of the anomalous and chronic intermittents, but still discernible ; while doubtless it may sometimes be wanting or difficult to make out, as is so often the case in such obscure intermittents. When most distinct, it is like the sensation of cold water applied to some part of the face, or trickling over it, being indeed often thus described by patients ; or there may sometimes be a sensation of cold, more general, if also transitory. The skin, at least of the face, also becomes pale and shrunk, with that peculiar physiognomy attending ague, so indicative of all these diseases, if so perpetually overlooked : and this is a symptom which, if unnoticed by the patient, ought never to escape the eye of an observing physician, explanatory, and often useful as it is. Occasionally, this paleness is local instead of general; and I have seen cases where I could pronounce that the paroxysm was threatening, from one side of the face turning suddenly white while the other retained its natural aspect and colour.

If this is the cold stage of this particular intermittent disease, the fit of pain appears to belong to the hot one, or thus at least has it always seemed in my experience: and if, as a hot stage, it is not a very marked febrile state, it is sometimes sufficiently apparent, in an increase of heat, local if not general, in the change of the pulse, and in a thirst which occasionally accompanies it. And if it is a slightly-marked hot fit, it is not slighter than that which often occurs in chronic intermittent when other kinds of local symptoms are present, or even when the cases are pure; while in such cases, as well as in this and other Neuralgiæ, the sweating stage is rarely well marked, or is perhaps only discovered by the facility with which that effect is produced by exertion.

Thus I have described a paroxysm of Neuralgia as resembling that of an obscure or chronic intermittent, with the super-addition of its peculiar pain or local affection of a nerve; and it remains to see what resemblance the general course of the disease bears to the common or simple intermittent, or rather, since I am not yet formally discussing the proofs of identity, what are the remaining characters of this disease.

It may be considered that there are two forms of this, as of other Neuralgiæ, the acute and the chronic; or the disease may be new, or habitual; a distinction which it is important to make, because the latter is, like all intermittents, far less regular than the former, while being perhaps more observed, it has aided in misleading physicians respecting the true nature of Neuralgia.

In the recent state, or in the first case, it returns in distinct and defined paroxysms, occupying a certain number of hours, or even, in some instances, but a few minutes, and leaving an interval of health. Where I have had the best opportunities of observing it, the returns are daily, or the type quotidian, while they maintain a regularity of period similar to that of common intermittent, and subject also to the same variations as to time. If I have also met with tertian, and even with quartan periods, they have been, in my own experience, more rare: nor, in these cases, has the disease been so apparently regular, though I have little doubt that the practice of others will here supply my deficiencies.

Now when the disease is of long standing, or of a chronic character, it is less distinguished by this regularity, and, further, as far as I have had opportunities of seeing it, is even more uncertain than the analogies, in common or anomalous intermittent, with which it is best compared; with the single exception, as far as I have seen, of the affection of the heart. Whatever may be the causes of this irregularity, if it is not the only fact which has aided in misleading physicians respecting the true nature of this disease, it is one which will probably cause many to suspend their judgments upon it at present, or to deny altogether the theory which I have here adopted. As an instance of these cases, I may quote one from Sauvages, where had it not been so durable, and not watched by an attentive physician, no regularity would have been perhaps suspected. In this, the pain occurred but once in eight days, yet never failing, and lasting thirty hours; while, being in the temporal muscle, it is called by Salius the *Hemicrania lunatica*. The entire duration extended to three years and a half.

I know not that I am able to explain why it should appear to be peculiarly irregular when compared to other chronic intermittents; yet if I fail, here or any where else, in elucidating difficulties, I must attribute it to a very narrow experience, arising from circumstances not worth detailing, and which I have vainly attempted to atone for by increased attention, finding little assistance from personal communication or printed records; so unsatisfactory are all these, from the fundamental deficiency of a system of observation founded on true views of the nature of these disorders.

As the matter stands, however, the first cause of obscurity must be sought in the rarity of the highly-marked cases and rigidly-local affections of this nature, which have secured for themselves the name of Tic douloureux. There is consequently a small number of cases from which to form an average and a judgment; whereas, were I to judge from all the cases of similar disorders, differing in violence and differing in place, which I intend here to rank under the same disease, I should decide that in the mass of neuralgic cases, the regularity was fully as great, under any type, as in the most acknowledged chronic intermittents.

Another ground of erroneous judgment as to this question, and which indeed I ought to have named first, has been the want of a correct original theory of the disorder; a want which, in science universally, is the parent of erroneous and deficient observation. In the ague, of whatever type, the most vulgar know the theory of this disease, and thus mark its regularity; while in this, where it is not known or expected, it is not noted, either by patient or physician: and accordingly, I have, in numerous cases, ascertained that a Neuralgia, before supposed uncertain, or never attended to, was found to be perfectly regular, as soon as either the patient or the physician had been informed that such was the character of the disorder. And it must be noted here also, that while a daily attendance on the part of the physician will scarcely happen in many cases, even among the opulent, while, with the lower classes, that is likely to be very casual, it is abundantly easy to conceive how the regular returns of a disorder, not previously suspected to be regular, should be overlooked.

There is yet another cause which has prevented the regularity of this disorder from being marked, where it is actually present: and it is an important one in some cases, as connected with the history and nature of Neuralgia. Even should the nerve be affected at the habitual period, that affection is not always a fit of the violent pain. On the contrary, it may be a very slender one, scarcely noticed by a patient used to much greater suffering; or it may appear as a toothach, or a headach, or what is called a rheumatic pain of the face: and thus, although really a paroxysm of the disease, may deceive both patient and physician, and most of all when, as in the cases last enumerated, it will admit of some common or vulgar name. Such in fact is the character of the Neuralgia, in whatever place situated: and the fundamental error here has been founded on that to which I formerly alluded, namely, the assigning it a name expressive of violent pain, and the separating this particular variety from all its analogies and variations. Such are the endless errors produced by ill-chosen terms, and by giving to such terms an improper value. And still further, as I shall hereafter more decidedly show, as this disease is but a mode of the intermittent, it may be exchanged for or replaced by a common paroxysm without any local pain: while even that paroxysm may be so slight as to attract little notice from any one, and at least of all from a patient accustomed to much keener sufferings.

Last of all, and particularly when of long standing, it becomes, like every other chronic intermittent, a truly irregular disease. Thus it may come as a single attack in the midst of weeks or months of repose, excited by some occasional cause; or it may, in the same way, adopt any modes of irregularity; which it is unnecessary to describe again, as it would be merely to repeat what I formerly said respecting chronic intermittents in their ordinary character.

Such then is the total character of the common Neuralgia of the face: and if I have dwelt on it at some length, this will render it less necessary to be minute as to its varieties, or as to other Neuralgiæ; since, the same general account will apply to all. Its similarity at least to intermittent, will even now be apparent, and very decidedly to those varieties which are attended by local diseases or symptoms; while the further evidences will appear in their due time. And if I might now treat of its cure, it will be best to defer this till I have enumerated all the other disorders of the same nature; since, with small exceptions, the same general rules will apply to all." 244—247.

From tic douloureux, he proceeds, in Chapter XI, to treat of periodical headache, and of vertigo.

That the headache in question, is a true Neuralgia in its pathological character, while ætiologically it is identified with intermittent fever, we are not at liberty to doubt. Indeed we do not think, that a better case could be selected, to show the connection between Malaria (or the cause of intermittent fever whatever it may be,) and *neuralgic* pain, (we know not how to avoid the solecism) than the one now under consideration. We have had, and still have, numerous opportunities of witnessing this distressihg disease, and can lend the weight of our humble testimony to the fact, that the pain is invariably of that kind which is denominated spasmodic, nervous or neuralgic—increased by blood letting, purging, and low diet, and removed or mitigated, by powerful stimulation. We have more than once known it mistaken for phrenitis, and have seen the conjunctiva of the eye of the affected side, engorged with blood, the eye ball tortured with violent pain, and the cheek overflown with a serous or lachrymose discharge ; but in the midst of all these *prima facie* evidences of inflammatory action, we have found nothing beneficial but *corroboration* Here then, we have abundant proof of its neuralgic character. On the other hand the evidences of its connection with marsh fever, are equally striking. 1. It occurs chiefly in the winter and spring, after intermittent fever has been prevalent in autumn. 2. It is almost confined to those who experienced attacks of that disease the fall before, or, sometimes, two years previously. 3. It is, commonly, most prevalent, when those relapses which are denominated vernal intermittents, are most numerous. 4. It has distinctly marked paroxysms, generally, in the Western country, quotidian ; is attended with various constitutional derangements ; and the fits continue, about the same length of time, with the fits of an intermittent. 5. It is curable by precisely the same means. Here we have, then, a well marked example of the connection between autumnal fever and neuralgia ; and on this alone we may raise a strong presumption, that many other painful maladies, the ætiology of which has not hitherto been understood, are owing to the same cause ; and thus prepare our minds for pursuing the inquiry, with candour, where ever our author may lead.

A disease so strikingly paroxysmal and maintaining so great an affinity with that under review, as the 'sick' or 'dyspeptic' head ache, could not escape Dr. M.'s observation, and accordingly he suggests, that many cases of it are in fact, but varieties of marsh neuralgia. We think this extremely probable. It abounds in the Western country, where the diseases from malaria are endemic ; we have observed it to be more prevalent in the valleys, than on the hills ; it often comes on independently of errors in diet, and frequently fails to return in the midst of them ; it is liable to be reproduced by exposure to cold, and the other causes which induce a relapse of intermittent fever ; its phenomena are almost the same with those of periodical head ache ; and the impending paroxysm can be frequently warded off by the same means that arrest the forming paroxysm of an intermittent ;—analagies more close and numerous, than have in many other *medical* cases, been thought sufficient to establish, at least, a generic identity.

Towards the close of the chapter under review, our author indulges himself in some animadvertions on the phrase—'a flow of blood to the head.' He thinks it would puzzle those who hold this language to explain their meaning. But we shall let him speak for himself.

"Moreover, in Neuralgiæ, every where, and in these headachs, there is an increase of the action of the smaller arteries, as I have formerly shown ; producing redness, or temporary and transitory inflammation, or else causing actual inflammation, as it does in the "rheumatism of the face :" and further, as I shall show hereafter, giving rise to a peculiar ophthalmia, a neuralgic ophthalmia. Indeed, even in a common headach, where the smaller nerves of the membrane are the seat of the pain, there is increased action, or excitement in the neighbouring arteries, as is well known ; extending often even to the larger ones, and even while the heart is unaffected and all the rest of the vascular system tranquil. So certain is it that undue arterial energy can be excited by locally disordered nerves, in a single spot, without fulness of blood, or "flow of blood to the head," or tendency to inflammation or to plethora, and even in subjects labouring under the most opposite conditions of exhaustion and debility. With this indeed, under various modes, physic is far too familiar to render it necessary for me to point out the circumstances under which it happens. Even in these cases, modern fashion is now resorting to this pernicious theory, pernicious in the practice to which it gives rise : when surely no physician really acquainted with disease, would consider that such partial increase of action in the vessels of the head, was a justification for blood-letting on the ground of a dangerous plethora or misdirection of the circulation.

What the exact nature of this increased local energy of the arteries is, any more than what it is when extending over the whole system in the common intermittent, we do not know ; so little do we really know of any thing in physiology or pathology. One fact here I may however add ; and that is, that in these local cases, it seems often to begin in the extreme vessels, and is from them communicated to the larger ones. But whatever it be, and whether in these particular cases it extends to the brain as it attacks the exterior small arteries, and even, as I remarked, the carotids, there is no evidence of its exciting inflammatory symptoms there ; though we can conceive this possible, at least under a certain form, since it produces this effect on the eye and the membranes of the mouth. If it were even thus, it is not a justification for blood-letting, but the reverse, as it will shortly be proved that all the neuralgic inflammations are aggravated by this treatment, as is the painful part of the disease itself under all its modes, and as is every chronic intermittent, be its form what it may ; while further, real diseases of the brain, or injuries to the nervous power, are here produced by blood-letting, as I have often noticed already, and shall have occasion to remark again.

But further, if this increase of action of the vessels of the head is suffered to take its own course, it subsides within a limited time, or with the local hot stage which produced it : maintaining, and proving still more effectually and completely, the analogy which it bears to the parallel symptom in the common intermittent fever. This would not happen in any other case of diseased, inflammatory action, either in the brain or in any other part : and it ought itself to be a proof that blood-letting is here unnecessary, (while it is in fact injurious) and that the common theory of increased action or flow of blood, true as it is in words, is false as it relates to science, in the view commonly taken of it, and pernicious as it relates to the treatment. And as far as it relates to the possible termination of this increase of action in membraneous or "rheumatic" inflammation, which is the only kind of inflammafion that does follow it, I may observe that this event is very rare, compared to the cases where it does not occur, and therefore diminishes the force of even the false arguments in favour of this practice that might be derived from it: while even that inflammation, where we can really prove its existence, is of a peculiar character, so as, almost universally, to be aggravated by evacuating and debilitating remedies, as I shall have occasion to prove fully hereafter. Such is the view that I have taken of this particular symptom or collection of symptoms, now called the "flow of blood to the head:" and if I have noticed it, as was here my duty, as belonging to the periodical headach or local neuralgic intermittent, it seems to me that nearly the same reasoning, with the same practice, applies to all the cases where these symptoms occur, whatever may be their original causes; since, in all, the pathological condition is similar." 264—265.

In conclusion, he speaks of vertigo as an associate of periodical head ache, though frequently occurring independently of it He thinks it often the consequence of the chronic action of Malaria, or of intermittent fever; and deprecates the practice of indiscriminate blood letting or cupping, which is resorted to, under the theory of sanguineous engorgement of the brain.

The XII Chapter, is on 'Neuralgiæ of other nerves of various parts of the body.'

"It might have been conjectured long ago, and with no very great effort of ability in the art of generalization, that if a nerve in the face might be thus diseased, so might any other nerve; and it does appear somewhat wonderful that Sciatica had not long ago led physicians to this obvious inference, at least for that particular case, had it done no more. And accordingly, if every individual nerve in the body has not been the seat of Neuralgia, it has occurred in so many, even in my own limited experience, that we have no reason to exclude it from any, but have, on the contrary, reason to expect, that whenever the disease shall become generally acknowledged and observed, there will be produced cases of its occurrence in every part of the body, and that such disorders will also be found not less widely and commonly diffused.

With respect to the evidence that the diseases which I am about to point out are truly Neuralgiæ, I may anticipate the further proofs that will hereafter be given, in a general summary, as also such particular ones as any of the remarkable cases here quoted may require, by saying generally, that they are of the same nature as those which have already been treated of on so many occasions. The quality of the pain is the same as in the one or the other of the analogous disorders occurring in the head; while there is neither organic disease, nor inflammation, nor gout, nor any of the other well known causes of pain present to justify it. It is periodical, or transitory, and recurrent, and through long periods of time, even through life: subject of course to those irregularities in this respect which belong to all the chronic intermittents; the nature and causes of which have already been explained. Very often we can trace it to the same causes; and while it is cured by the same general remedies as common intermittent, with the addition of such local ones as are found of use in the Neuralgia of the face, it is exasperated or rendered permanent by that which is equally maltreatment in all those diseases: while also the very pointed evil consequences, produced by the evacuant and debilitating practice, are the very same or exactly analogous to what they are in Intermittent and in acknowledged Neuralgia, whatever be the place of the pain. Further, these pains, or painful disorders, be the situations what they may, alternate with common Intermittent, in the several ways already described as occurring in the local anomalies of that disorder, and in Neuralgia and periodical headach; that is, as whole periods, or as mere paroxysms: while, of course, they are also irregularly intermixed with such affections, as happens with respect to the whole of the disorders treated of in this essay, whenever they are of long duration, or have become an inveterate habit." 269—270.

The Optic Nerve. Our author has met with one case of this Neuralgia. The patient described the pain 'as if a red hot needle had been passed deeply through the centre of the eye.'

The Testicle. Of this he has seen two cases. In one it was mistaken for scirrhus and the gland extirpated. It was found healthy in structure; and the disease returned in the spermatic cord. The other case was associated with the slight paroxysms of an intermittent. It was cured by arsenic.

The hands and fingers. Dr. M. has met with many cases of this variety. We shall extract his account of one of them.

"Another case has been mistaken for gout, the pain being seated in the knuckle joint of the little finger; while what renders this instance remarkable, there seemed no intermission, or thus at least the patient positively asserted, during a month through which it lasted. It was complained of as very severe, and as occupying the space of a pea, while attended with the usual sensibility of the surrounding skin, but without swelling or redness at any time. This ought, negatively, at least, to have indicated its real character, nor shall I be surprised to hear of many more such cases when this subject shall be better understood. In this instance also, I had an opportunity of remarking the evil consequences of blistering; nor could I indeed have avoided that, since the mistake, like the practice, was my own; this somewhat obscure case having occurred in the earlier days of my investigations on this subject, and long before I was aware of its extent, or of the various appearances of Neuralgia. That I did not cure it by my practice, I am bound to add: but having instantly, in a minute in fact, disappeared on the occurrence of a periodical toothach attended by an intermittent, which I doubt not I had previously overlooked, its real nature became evident; while numerous similar terminations since that time, have left no doubt in my mind respecting its real nature."
274.

The Knee. The profession are familiar with the sympathetic neuralgia of this joint, which attends diseases of the acetabulum. A similar affection is sometimes connected with fever, and ceases when the malady on which it is engrafted, is cured by the usual means. Our author cautions the profession not to mistake neuralgia for scrophula of the knee joint. This affection has often been denominated rheumatic and gouty. Dr. M. has seen one case of five years duration, complicated with visceral disease, which yielded to the bark and arsenic given alternately. In two other instances 'the extremely acute pain was situated immediately over the very edge of the head of the tibia' and the affected part was not more than an eighth of an inch in diameter. There is in this part he observes, no *visible* nerve, and hence we are made to see, that a neuralgia the most intense, may exist in a nerve too small for anatomical exhibition. In another case, 'there was, one day, a pain in both the knees, and, on the alternating one, a pain in one arm,' in which type it had continued a long time, and was called an irregular gout.

The Tibia. Of the neuralgia of this bone, or rather of its periosteum, our author has seen cases which alternated with neuralgia of the head, and has, also, met with others, in association with well marked chronic intermittent. After stating these facts, he throws out the intimation, that 'nerves are more subject to neuralgia as they approach nearer to the surface of the body'—a suggestion to which we invite the attention of our readers.

The Toes.

"In one instance," says our author, "I have met with a highly marked case of this nature in a toe, arising from, or connected with, an obscure intermittent accompanying a morbid spleen.

The pain itself was limited to the very end of the toe ; but, after some duration, an uneasy sensation, accompanied by numbness, extended along the branch and trunk, even into the sciatic nerve at the hip ; while, even when that extended pain had not come on, the patient felt a similar sensation on my attempting to trace the course of these with my finger, along the surface. The cure of this case proved somewhat difficult, though it was at length removed by the means already mentioned ; and while for some time it increased in severity, it at length happened that the uneasiness and numbness, after reaching the hip, reappeared in the shoulder, passing down through the ulnar nerve, and affecting the end of the finger of the same side which corresponded with that toe, or the ring finger." 277—278.

The Rectum. Of neuralgia of this part, Dr. M. has met with a striking example, which we shall quote in his own words.

"At the first attack, and for some weeks, it consisted in an occasional sensation like a spasm, apparently situated in the urethra, about the prostate gland ; recurring three or four times a day, and causing little uneasiness. Gradually, these sensations increased in frequency, and were attended with a general sense of irritation about the neck of the bladder, very much increased by walking, and at length producing spasms in various parts, with a tendency to an hysterical paroxysm. No apparent fever of any kind was at first present ; nor any suspicion of its real nature entertained ; while the disorder, not yet strictly periodical, was referred to the urethra and bladder. Very shortly, there supervened a debility, with occasional numbness, in one leg ; and it was easy to trace, by the tingling sensation formerly described, the course of the fibular nerve. At the same time also, it was perceived that the mere act of bending the neck forwards, brought on the sensation in the perineum, and further, caused the patient to totter on the affected leg ; a circumstance to which I shall have occasion to recur hereafter.

After enduring some weeks in this form, there supervened a febrile state, at first very obscure, but which was, after some time, ascertained to be a double quotidian with a nocturnal and diurnal paroxysm. Still, the nature and the real seat of the pain was obscure ; nor was the slightest suspicion of Neuralgia entertained by the numerous medical attendants who in succession examined the case. Such however it appeared to me ; and after some further progress, every doubt in my mind, if not in that of others, was removed by the increased regularity of the disease, and by the pain in question becoming as regular as the attacks of Neuralgia are when most perfect. In this state, the first of the quotidian paroxysms was simple, and the second was attended by the Neuralgia, which, now increasing in decision, increased also in severity. In this aggravated state also, it became plain that the primary seat of the pain was in the rectum, the patient describing it as a burning heat, as from a heated solid introduced, which was shortly communicated to the bladder, producing irritation and strangury. When of this severe nature, that irritation extended even round the thighs and over the lumbar region ; so that the slightest touch produced great uneasiness, as happens in violent cases of the Neu-

-algia of the face, and was felt even in the abdomen, as if the whole colon was affected in a similar manner ; which was probably the fact.

Further, during the severity of the attack, all the limbs were affected with spasms ; and very generally there supervened a regular fit of hysteria, with a great degree of general derangement throughout the whole system, consisting of the usual symptoms of a severe remittent in all their worst forms. Lastly, and not to be unnecessarily minute, as the irritation of the bladder appeared to spread along the ureters to the kidneys, there came on diabetes, the diabetes mellitus ; while, when this symptom was peculiarly severe, it was attended with an acute pain in the left, but not in the right kidney, so that possibly this particular symptom was confined to one of the glands only. And respecting this part of the disease, I must further add, that it was rigidly paroxysmal, or that the morbid secretion of sugar commenced with the general fit, and entirely disappeared in the interval." 279—280.

The Thigh. Our author has seen the 'anterior cruval nerve' the seat of this malady, in different individuals. It existed in the trunk of the nerve, at the exact flexure of the thigh, and in its ramifications, as low as the middle of the limb, 'shooting as sciatica does, and with similar severity, down even to the toes.' After speaking of some of these cases he adds—

"And in another of these cases, where the disease was similarly at the flexure of the thigh, it proved extremely troublesome : recurring during many years, sometimes superseding another Neuralgia, at other times accompanying a marked intermittent of no small severity, while removed either by the usual remedies, or by change of place, or by the supervention of some other mode of the disease." 284—285.

The Kidney. It has occurred to our author to see one case of 'pure pain' apparently in this organ. It was attended with no diabetes, and existed he supposes in the renal nerve.

Chapter XIII, is devoted to Sciatica, which has so long been recognized as a distinct and serious malady, that our author thinks it entitled to a separate consideration. After several paragraphs of criticism on the views which preceding writers had taken of this affection, and expressing the opinion that it should be regarded as a true neuralgia, Dr. M. observes—

"It is on this view of its nature, that we can explain the peculiar intensity of the pain ; a pain, both for quality and violence, bearing no resemblance to that of rheumatism. On this view also, we can explain its confined nature, the absence of inflammation, the state of diffused irritation about the parts which attends the fit of pain, the shocks which accompany it, resembling those of all the Neuralgiæ, and its propagation along the course of the nerve. And if these peculiarities are thus explained, so are they the proofs of its real nature ; while similar and further proofs are found in the paralytic affection which so often follows it, and which, as I have already shown and shall more fully show hereafter, is a frequent consequence in all Neuralgiæ, particularly under bad or wrong treatment If spasms in the adjoining muscles, or throughout the body in general occur in sciatica, so do they in all the Neuralgiæ ; and it is further remarkable that it produces, in irritable habits especially, hysterical symptoms, as do the other disorders of this nature, together with a general irritability, both of mind and body, which seems to belong peculiarly to the painful diseases of this nature.

It is further remarked that the pain of sciatica "breaks down the constitution;" or that there is a state of general disorder, in the chronic cases especially, which is scarcely produced by any chronic painful disease, not even by gout and stone. This is the condition or change to which I formerly alluded and shall have occasion to allude again, and the cause of which I then explained; a condition which attends all the Neuralgiæ without exception, when of long continuance. If other proofs of similarity or identity were required, they would be found in its inveterate duration when once established, an inveteracy which has rendered it an incurable disease; in the inutility or the mischievous effects of blood-letting and local applications; in its apparently voluntary cessation on change of place or of habits, and in other circumstances, attending the remedies for good and evil, which I need not again repeat, often as they have been discussed for the parallel cases." 290—291.

After dwelling for a short time on the difficulties which are presented to him who may attempt to class this disorder with those from Malaria, he remarks—

"Thus does it appear to me that the character of sciatica is perfectly established as a Neuralgia, or as coming under the division of local or neuralgic Intermittent; and how important this view is, as to the practice in this disorder, a practice which has hitherto disgraced physic by its failures, I need not say. I do not mean at the same time to assert, what in fact I do not suppose, that it is necessarily the produce of Malaria; because I have already shown that the other Neuralgiæ appear to arise often from simpler causes, and are assuredly the produce of even local injury in some cases. If in attempting to ascertain what the common nature of any diseases may be, or in arranging any number of varieties under one common genus, or even one common proximate cause, we are to be called upon to find a common remote or exciting cause, this is not the only case, by many, in which we should have to dispute our way through nosological arrangements. Even simple intermittent may, for aught that we yet know, have more causes than Malaria, and it is, perhaps, even probable that it has; and thus may the Neuralgiæ, and the sciatica among them, be the produce of mere cold or damp, as the latter disease has been commonly supposed to be." 292.

The 14th Chapter is appropriated to "Questionable of Neuralgiæ," in which the author briefly enumerates several painful affections, which, he thinks, further and more acurate observation will class with the neuralgiæ. We shall pass them by.

The next Chapter relates to 'Neuralgic affections of the Glands.' It is equally short and unsatisfactory. He thinks the lachrymal gland is sometimes affected, and likewise the salivary; but attaches more importance to the idea, that the secretion of bile and urine, is often influenced by a neuralgic condition of the hepatic and renal nerves; and, even, intimates, that diabetes mellitus, may hereafter be found connected with this condition. The facts, however, are, as yet, but few and equivocal, and, contenting ourselves with recommending the subject to the attention of our brethren, we shall proceed to matters of greater certainty—the Neuralgiæ from injuries of the nerves. These make up the subject of Chapter XIV.

Dr. M. complains of a want of facts to illustrate this variety of neuralgia. He has, however, cited several of an instructive character, and the experience of every practical man might furnish others.

If a nerve be divided with a cutting instrument, a neuralgia is not likely to ensue, for incised wounds and amputations seldom produce it. It is the puncture, laceration, contusion, compression, torsion or stretching of a nerve, which is, generally, followed by neuralgic disease. This is sometimes in the part affected, at others above or below it. A considerable degree of paralysis is often the consequence. Sometimes, at a remote period, a paralysis agitans ensues, or, about to establish itself in the system, commences, in a part formerly injured. A reduction of temperature and feeling, with a decay in the size of the limb or joint affected, is a frequent consequence of injuries of this kind—of which we have seen striking examples, in the hip, knee and ankle joints. In some instances, the injured part swells and becomes considerably indurated, but does not suppurate. We have, at this time, a patient affected with neuralgia of the great toe, which is nearly twice the size of the former. It was caused by a bruise, and has lasted three years. When the patient is dyspeptic the pain is generally much worse. At times it is replaced, by gastrodynia, or neuralgic pain in some other part. In some persons, there seems to be a predisposition to neuralgia, when it follows the slightest external injury, and occurs in various parts of the body. We have met with several examples of this kind, some of which were, clearly, the consequence of autumnal fever; affording additional evidence of a relation between that disease and neuralgia. Our outhor has met with cases of this malady which assumed a distinct quotidian character. We shall extract one of them—

"In the next case, the injury was a blow on the arm, producing a severe bruise, a fact however which was only discovered by cross-examination, as a regular Neuralgia of a quotidian character was established long before I saw the patient, who was also a young woman. In this instance also, I was led to inquire respecting previous injury, from having long suspected that it was not a very unfrequent cause of Neuralgia; and the information was satisfactory, inasmuch as the disorder was distinctly stated to have followed the bruise after two or three days. It was remarkable however that the seat of the neuralgic acute pain, which was in the middle of the radical nerve, and which extended in the usual manner to the shoulder, was not at the place of the injury, but considerably distant from it. While under cure, this case of Neuralgia lasted long enough to enable me to ascertain personally its perfectly regular character, and, as in the preceding case, that cure was effected by arsenic." 308.

The excessive pain attendant on Corns, is manifestly neuralgic, as well as that produced by certain ulcers. The former never occasion suppuration, and in the latter, the secretion of pus is always imperfect, when the surrounding parts become neuralgic and irritable.

Tooth-ach, is the subject of the next Chapter, which runs through nearly 40 pages. In the opinion of our author, 'every tooth-ach is a neuralgia, be its forms whatever they may.'

"The heads under which toothach is divided, may, for the present purpose, be conveniently rather than correctly, assumed as the following : but they are very indefinite divisions, as they run into each other, and, as individual cases, present endless modifications.

It may be rigidly periodical : while, in this case, it may be either combined, or not, with chronic intermittent fever : and further, in this case also, a tooth, or teeth may be sound, or carious, this condition being here considered incidental, not essential. Or, it may be irregular in its paroxysms, under all the same variations ; and in both of these varieties, it passes into the common Neuralgia of the face ; or, cases occur in which either name is equally applicable. This is the periodical toothach so familiar to those who have paid the attention to this subject which it deserves, but which is so very often overlooked, not merely by the physician, but very generally, if somewhat more remarkably, by the patient ; as, by the former, its passage into highly marked Neuralgia seems equally to have been neglected, and most so when a carious tooth is present.

In these forms, it is here considered as spontaneous Neuralgia : while, in the next place, it may occur, either casually, or more regularly, that is, under all the variations of that general disease, and traceable in a marked manner to a carious tooth ; the pain being fixed in the nervous branch by which that is supplied, or originating in that and spreading from it. In this form it is here referred to Neuralgia arising from an injury to a nerve.

Further, as happens in cases of common Neuralgia, the excitement of the minuter vessels may be such as to produce temporary and paroxysmal, or, more permanent inflammation in the membranes, or generally about the face ; when it becomes rheumatic toothach or rheumatism of the face, (neuralgic inflammation.) The chief varieties here, are produced in the following manner. The general (rheumatic) inflammation may, as I have just suggested, be paroxysmal and periodical, and even regular, or it may be permanent ; while it may attend a carious tooth which is pained, or free of pain, or else a sound tooth in a state of pain ; and such pains under any mode of regularity or otherwise. It may also be accompanied by a neuralgic pain independent of the general pain of the inflammation, and not in a tooth ; in which case, it is plain, it belongs most unquestionably to Neuralgia, though, in the usual practice, ranked with toothach.

Or, lastly, all other pain but that of the inflammation itself may be wanting ; when it becomes, most strictly, what is called rheumatism of the face : this state also sometimes occurring, even without the presence of a carious tooth. If this rheumatism of the face with separate neuralgic pain should in correctness have been ranked under common "Tic," the last division, also, in a more scientific arrangement, should have been reserved for a distinct section : but its obvious affinities and the received usage render it convenient to include it in the present one ; particularly as its limits with respect to the former varieties are indefinite." 319—320.

After dwelling for some time, on the causes which introduce great irregularity into the attacks of tooth-ach, our author proceeds to trace its analogies with neuralgia and intermittents.

"In numerous cases of common toothach, in every well marked and severe one at least that I have ever had proper opportunities of examining, I have never failed to trace one or more of those symptoms which attend simple intermittent ; that is, indications, varying in number and degree, of a febrile paroxysm : and while those symptoms have been, in general, particularly, discernible in the chronic cases, or where toothach had become an often recurring habit, I have found ample reason to believe, that, wherever the disease has persecuted the patient through a long life, the visceral glands have been affected ; or, that however irregular, it has, like the irregular and chronic intermittent, been associated, whether as effect or coincidence I have already declared my uncertainty, with derangements of the liver or spleen, or both, but most frequently of the latter. And in some cases, this connexion has been so obvious, that while the patient was habitually suffering under a palpable or a grievous chronic intermittent, the irregular toothach has been associated with the disease in all the ways in which the periodical one is, or in the exact modes already pointed out as to the several Neuralgiæ.

And I have here a collateral remark, which is of some importance; while I alluded to the same fact as to Neuralgia in general, on a former occasion. It is frequently mentioned in medical writings, that the pain of toothach, when recurring severely for many years, destroys the constitution; and this effect has been attributed to the severity of the pain. The constitutional disease here alluded to, is, doubtless, great; but, as I formerly hinted, it is not produced by other pains, nor is there any thing in pure pain to account for the species of injury in question. This is one of the errors, far too common in physic, which flow from a minute or petty attention to an obvious symptom, to the neglect of generalization. In these cases, the constitutional disease in question will invariably be found to be a chronic intermittent, sometimes united to visceral affection, and to be thus, rather the cause of the toothach than its effects; the former being, at least the great disorder, of which the pain is but a portion or a symptom. And should the toothach destroy a patient, or shorten his days, as it is said to have done, it is easy to see to what causes such effects are to be attributed; while such erroneous and trifling judgments in cases of such a nature as this, are even more unpardonable. But I must withdraw from a criticism which, did I pursue it, might involve a censure on many to whom I must look with respect on other points, however they may have suffered themselves to be misled on this one, by following, from habit, minds inferior to their own.

The general nervous affections which attend common toothach, are the same as those which occur in all the Neuralgiæ, and, similarly, in all the intermittents, whether simple or anomalous. It is remarked, even to familiarity, that the pain of toothach is peculiarly intolerable, or affects singularly the whole body, and the mind also; while, as far as pains can be measured or compared, it is not equal in quantity to that of wounds, or of various painful diseases, which do not equally affect the system, or are not equally intolerable and not thus productive of irritability and impatience. Under many severe pains, the mind retains its full powers; and when the pain ceases, the inconvenience is over; while the former is rarely the fact in the toothach, and still more rarely so in the habitual one. This is precisely what occurs in every case of Neuralgia, be it whatever it may; and hence do the general sufferings, often undescribable, from periodical headach, Tic, or sciatica, resemble those which occur in severe toothach.

It surely must have been remarked by every one, that the physiognomical expression of a person labouring under toothach is peculiar, and unlike to that which occurs from gout, or the pain of wounds, or almost any other pain. Yet it is the same as that which takes place in Neuralgia, as an artist at least will immediately discover; and what is more, it will be found to resemble, in an air of peculiar anxiety and of melancholy, united or separate, as also at some periods, in a paleness and shrinking, and a peculiar greenish yellow or singularly sallow colour, that expression which characterizes the fit of intermittent, as I have more than once remarked respecting the Neuralgiæ in general.

To the peculiar situation of the pain, in the body or substance of a nerve, and communicating with the whole nervous system, we must probably look for these peculiarities, whatever the exact cause may be; and thus the toothach is also often attended by hysteria, even amounting to a fit, by lacrymation, or a tendency to it, and by a species of debility of mind, equally remarkable in every Neuralgia, and, in fact, in all the intermittent diseases under whatever form. Where dyspeptic and hypochondriacal symptoms occur in patients subject to habitual toothach, as is by no means unusual, the explanation becomes thus easy: and thus also we can explain, how it so often happens in females, that this disorder, when frequent and durable, is attended by menstrual derangements. In reality, in every patient subject to frequent and severe toothachs, there is a great deal of general disorder of various kinds; and it can always be traced, with due care, to a separate, if, in a certain sense, connected constitutional disease, which is that variety of remittent or intermittent amply described in former parts of this essay.

There is yet one familiar fact occurring in toothach, which also connects it with Neuralgia and intermittent; and though it may appear trifling as a proof of identity, it is too remarkable to be passed without notice. It is removed by charms, a practice not uncommon in remote parts of the country: and thus it is also that the sight of the operator or of the preparation for extraction, often puts a stop to it, and when not habitual, even permanently. Thus also it is frequently cured by change of place or habits, as it is removed by the occurrence of another disease or pain: remedies, all of them, which occasionally, and perhaps as often, produce the same effects in other Neuralgiæ and in intermittents."

If, occasionally, the pain is confined rigidly to one tooth, a fact, however, which occurs chiefly where the tooth is carious and the disease is the produce of local injury, it is much more common for it to extend beyond that. Thus, like the Neuralgia elsewhere, the severe pain will be in one point, of greater or less dimensions, while a more moderate pain extends along the courses of the nerves, even to great distances. And in some cases, where the chief pain is not particularly pungent, the secondary one may be such that the patient can scarcely say where it really does lie; while, further, it is subject to wander about the face, or to be most intense at times in one part and at others in another. Thus does common toothach, like Neuralgia, often pass into headach; as, further, the patient may be so equally occupied between headach and toothach, as not to know precisely by what he is most grieved. In such cases as this, nothing but inveterate habit and inattention, added to the weight of a name, to that which is always the most weighty argument to the multitude, could prevent practitioners from referring the disorder to Neuralgia instead of toothach; had they indeed known what Neuralgia itself was.

It is not uncommon for the disease, the Neuralgia, to be situated in one or more of the leading branches which supply, by their minuter ramifications, a number of teeth. In this case, the pain may actually extend into many teeth, or to one half of a row, in either jaw, or in both jaws, or to the whole row, in either, or even in both jaws; though cases where it occupies more than the whole of one row are rare, while this accident seems to occur more frequently in the upper than in the lower jaw. It may also, in this case, affect but two teeth, or even one; or, in such a case as the last, there may be a neuralgic pain of the cheek or face, with a decided toothach superadded, in a single tooth, which, in the present inquiry, I shall presume to be sound."

I showed in a former part of this essay, that the local injury of a nerve could produce a regular periodical Neuralgia; while I further suggested, what indeed is almost obvious, that the irregular pains produced by such a cause ought also to be considered of the same nature. Now these are cases exactly analogous to the toothach from a carious tooth; and thus we can see, how, from such a cause, even a periodical toothach might be produced. The extremity of the ramification supplying the tooth is, if not an injured nerve, an exposed one, susceptible of occasional injury, and also, it is probable, of inflammation: while it is not very improbable that the exciting disorder in the nerve in every case, the permanent cause which produces occasional fits of pain, is, if not a species of inflammation, something analogous to it. Hence, if the prick of a needle in the finger, or any other injury, can excite true Neuralgia, in the same or in a distant part, as I have shown that it can do, it is easy to imagine how, not only simple toothach of the part, but even Neuralgia in the face, may be produced by a carious tooth."

Carious teeth do not invariably produce toothach, far from it; even when we are sure that the sensible parts are equally exposed, and when the proof of this is perfect, from the pain which an injury, or the contact of a stimulating substance produces. So far from that, we find thousands wasting away through years, and with the sensible part exposed the whole time, and yet without exciting pain ; while, when excited, even violently, by accident or design, for a few minutes or hours, it terminates, not to recur again but from fresh injury. The most common reasoning should therefore show, that the disease does not essentially consist in such exposure or injury, but that something else is present, and that this is but an occasional cause. That fundamental cause lies in the nerve, or the nervous system, as in all Neuralgiæ." 330—345.

These extended extracts will sufficiently make known our authors views. He proceeds now to treat of the cure of this variety of Neuralgia, but our limits will not admit more than an abstract of the cure of neuralgia in general, at which we shall presently arrive.

In the conclusion of the Chapter, Dr. M. speaks briefly of rheumatism of the face.

" I formerly showed that it was a frequent effect of Neuralgia to excite an action in the minute arteries, tending in some cases to inflammation, and as I shall hereafter show, sometimes actually producing it. I have further compared, and must for the present purpose compare, again, that action of the arteries in a part, to the effect which takes place, of an analogous nature, in the whole arterial system during the paroxysm of intermittent.

Supposing a carious tooth to be present, there are two modes in which the rheumatic inflammation may occur. If the pain has commenced in the tooth, the inflammation may be determined or produced by that ; and we may then conceive it as the produce of the Neuralgia in the main nervous branch supplying the tooth ; an excess of that action which so generally occurs in the neighbouring vessels in every Neuralgia. But the rheumatic or inflammatory action may also be the prime or leading disorder, while the pain of the tooth is produced by its extension. In this case we may compare it with the local rheumatisms formerly described as connected with intermittent, of which it is in reality a frequent variety.

If, on the other hand, there is no carious tooth, the rheumatic inflammation in question bears still more strongly the marks of an original disease, while in this case, its characters and origin are those just described. And here, if it is most generally a simple disease, a merely painful state of the membranes of the face, attended with inflammation and swelling, or not, it sometimes also excites a distinct neuralgic pain in addition, or else appears to originate in one ; such cases being exact parallels to those just described where a carious tooth is present." 346—347.

With these copious extracts, we had intended to close this part of our analysis, but are tempted to transcribe a condensed view of the whole argument of the chapter, inserted near its close ; inasmuch, as it presents the subject in a clearer manner than the preceding paragraphs, and shows that our author *is* capable of writing with conciseness.

" Neuralgia is a pain occupying some point in the nerves of the face, among others ; and it may occupy any point in any large branch which supplies the teeth, among other nerves of the face. The pain which it produces is the same pain, whatever be the nerve or part of that nerve affected. The pain of toothach is the same pain, and it is seated in an ultimate extremity of the branch which supplies the teeth, or in more. If that pain is not Neuralgia, then it must follow, that although every other point of that nerve, when pained, is suffering from Neuralgia, let that pain exist any where, from the brain even to the extremity, the very last, ultimate, point thus suffering, suffers from a different disease. Reductio ad absurdum.

Neuralgia is regularly intermittent and periodical, or it becomes irregular under various modes, and from causes, of which many are assignable. The same is true of toothach. Neuralgia is often, or perhaps generally, attended by a peculiar constitutional affection, ascertained to be a chronic intermittent, and when highly marked, there is a febrile paroxysm accompanying the pain. All this is true of toothach. Neuralgia alternates with intermittent fever, by relapses; so does toothach: it alternates with it by paroxysms, and toothach does the same. And in both, the types may be quotidian, tertian, and double tertian, or perhaps more; while, in both, the disease may be similarly doubled; or, being a double tertian, may consist of a paroxysm of the pure intermittent on one day, and of the Neuralgia, or of the toothach on another.

Neuralgia is attended by heat, and by excitement of the minute arteries, accompanied by general diffused pain, or irritability, or both; and so is toothach. Neuralgia and toothach are united, or simultaneous; or, the pain, and the place of the pain may be such, that neither the physician nor the patient can determine what the disease ought to be called. Neuralgia passes into, or produces toothach; and, reversely, toothach passes into, or produces Neuralgia. The two pains alternate, in various modes; or that which was Neuralgia at one period, be that of day, hour, or minute, or even instant, may be toothach in the next; or the reverse.

Neuralgia is produced by the injury of a nerve. So is toothach; and this is the case of a carious tooth. And if toothach from this cause is especially frequent, it is that the caries of a tooth is very common; and that there are not, in external circumstances, or in the body, any frequent means of thus injuring, either through accident or disease, the branch of a nerve elsewhere."
350—351.

From Neuralgia of the teeth, Dr. M. proceeds, in the 18th Chapter, to neuralgia of the eye—which he denominates neuralgic ophthalmia, and rheumatism of the eye. His leading object is to prove, that what is called rheumatism of the eye, is at once neuralgic and miasmatic. The following is his description of this malady.

" There is a peculiarity in the aspect of the inflammation itself, far easier to recognise than to describe, and by which alone it is generally distinguishable, even at a distance, and on a mere glance, to those who have acquired that experience which in other cases is called the *tactus eruditus*. I have sought in vain for expressions to say fully what this is; but I believe it to be as useless as difficult, since, however accurate they might appear to those who already know this inflammation by sight, they would not teach others to know it, inasmuch as no visible object can be justly described to the previously ignorant; while such a description would be useless to those who are already experienced in this ophthalmia. The more obvious character, however, is a dull, rather than a lively red colour, not unfrequently attended by a tinge of yellow; the cause of which is especially visible in the sound eye when but one is inflamed, and the source of which must now also be obvious, particularly in autumnal cases. This inflammation occupies the whole conjunctiva, even to the verge of the cornea; and while the redness is rather produced by the minutest branches of the arteries than the larger ones, the general aspect is almost that of an additional coat of red cloth in the severer cases, sometimes attaining a higher level than that of the cornea.

In severity, however, it differs exceedingly from a mere general, and somewhat pale redness of the conjunctiva, to that violent inflammation just noticed. Here, it is apt to resemble the celebrated contagious and purulent ophthalmia; but it can nevertheless be distinguished by attending to its progress and to the collateral symptoms, while it never, as far as I know it, suppurates on the surface, like that disease. This is a part of its history, however, on which I must yet speak with some hesitation; as, after many years of observation, whence I concluded that it never did suppurate, my opinions have been recently shaken by one or two cases, though I had not the opportunity that was necessary for satisfying myself as to the real nature of the disease in these. Whenever it shall, as a separate disease, have received from physicians the further attention which it requires, this, and some other circumstances, which I cannot now well elucidate, will be better understood, while I shall gladly avail myself of such information : though it will be necessary that this disorder shall be truly discriminated for this purpose, lest we return into worse confusion than that which I am attempting to rectify.

Such is the general and obvious character ; while I shall reserve some rarer particulars for consideration immediately But there are one or two remarkable circumstances respecting it, too characteristic to be passed over. It is often unattended by any pain in the eye itself ; and this seems particularly the fact where it is of long standing, and not very severe ; though there are cases also in which it is accompanied by as much pain and irritation as the common, or even the contagious ophthalmia. And in such mild chronic cases also, there is, sometimes, not even irritability to light ; so that, with the exception of the occasional feeling of dust or sand, it will often last for many months as a mere deformity ; the patient, if in low life especially, not applying for advice. It is also a peculiarly obstinate species of ophthalmia ; continuing unchanged, even for months, in spite of every ordinary means of cure, particularly resisting local applications, and, as I shall presently show, very commonly aggravated, and in a very marked manner, by the evacuating system. Wardrop has also remarked that there is, at the commencement, a peculiar sense of dryness in the eye, while that is at length followed by abundant lacrymation. If I cannot say that I have very decidedly noticed the first of these symptoms, it is probably because of a well-known usage, in consequence of which, physicians rarely see the commencement of a disease, as, with respect to the lower orders, is true of almost every member of the profession ; but I can bear abundant testimony to the lacrymation, while its exceedingly remarkable nature is explained, to me at least, on those principles already stated in speaking of the neuralgic affection of glands.

When there is no pain in the ball of the eye, it would seem that the conjunctiva alone is affected ; while, when irritability to light attends, we must suppose that the neighbouring vessels, and nerves, within the eye are in that state, be it from sympathy or extension, which so often occurs in the rheumatism of the face and in common Neuralgia, where, added to the decided inflammation and pain, there is an excitement, a tenderness, or an irritability in the adjoining parts. It is not necessary that the eyelids should be affected, or that the inflammation of the conjunctiva of the eye should extend over that of the eyelid ; though this happens in the severer cases, and, as it would seem, rather in the acute than the chronic ones. It is a fortunate circumstance, that this inflammation is so much and so often resisted by the transparent cornea, as is the fact also in some other ophthalmias, but abundant instances of this do nevertheless occur rigidly speaking, and in the severer cases, the cornea becomes dull ; and if this opacity proceeds, it at length forms a cloud or a spot which diffuses itself over the whole eye, while it is more condensed in the centre. Fortunately, even when very considerable, this commonly disappears, under proper treatment of the general disease, and even within a day or two ; while I have seen it return many times, under different relapses and in successive seasons. without any more permanent effects. In such cases, also, it will sometimes be found, by means of a lens, that there is an ulterior disorder of the cornea, resembling very superficial ulceration ; equally disappearing, and without bad consequences, with the general inflammation. Far more rarely does it affect the iris ; but cases even happen, as I shall soon show, where that membrane alone is the seat of the disease ; the neuralgic affection producing here a rheumatism of the iris ; to adopt the common phraseology.

Supposing that the inflammation, thus complete or severe, has not been cured, or, as is the fact, has been neglected or maltreated, the ulterior unfortunate terminations may be as follows : In the acuter cases, the ulceration of the cornea may increase so as to perforate it, in which case the eye collapses, commonly for life ; though this is sometimes healed, with a partial or perhaps entire restoration of the figure at least of the eye-ball. In still worse cases, matter forms within the globe of the eye, and it bursts; and I much suspect that the cases of single eyes thus lost, which are not uncommon, might all be traced to this particular disease. In what may be considered acute cases also, if less severe, a pustule, or minute abscess, is sometimes formed in the cornea, inducing, of course, blindness; and further, in these, this portion of the eye will become permanently opake, while opacities are the frequent terminations of the chronic cases, misunderstood and maltreated. I shall be much mistaken if the very great majority of cases of opacity in one eye, as well as not a few where both are thus affected, have not arisen from this, the most common assuredly, of all the varieties of ophthalmia, though so little suspected, or rather so nearly unknown: since the essay of Wardrop, correct in the practice which it points out, seems to have produced little effect on the mass of practitioners; while if it had, from not distinguishing the chronic forms, it leaves those persons still in the dark as to the predominant cases of this ophthalmia.

The neuralgic ophthalmia sometimes attacks suddenly, and appears within a few hours, in all the perfection which it is about to preserve; but it is often also preceded by an intermittent or remittent febrile state, sometimes so slight as to be overlooked, not only before the disease, but during its continuance. And, as far as I have observed, some symptoms of this are always present; though it is very seldom that we can ascertain that it has preceded, because the patient has paid no attention to a disorder so slight. On other occasions, it is the sequel of a previous distinct neuralgic pain, which sometimes lasts even a long time before the inflammation comes on. That neuralgic pain is also, as the precursor of this ophthalmia, most commonly seated in the face, and very generally in the eyebrow or the temple, though sometimes even in the lower jaw; being, in many cases, a common hemicrania, or a periodical head-ach of some kind, or a clavus; though I have seen cases also, where it has been situated in even more remote parts, such as the arm. Further, this previous disease will occur as a rheumatism, in perhaps any part, or even as general or diffused rheumatism: the ophthalmia following it, perhaps being continued from it, or else appearing as a replacing disease. All these circumstances mark distinctly its connexion with intermittent and with Neuralgia in general: and when it replaces another rheumatism or another Neuralgia, this is precisely what occurs so often in the anomalous intermittents as they were formerly described." 358—361.

We have met with many cases of the disease here described. A recent instance may be mentioned as an example of the whole. In the winter of 1828–9, a man applied, at the Cincinnati Eye Infirmary, with ophthalmia. Both eyes had a uniform redness, with but little discharge or tumefaction. It had come on without any obvious cause. On inquiry it was found that he had experienced an attack of ague and fever in a neighbouring town, the preceding autumn. It was found necessary to resort to the bark. In 10 or 12 days the complaint vanished, leaving no trace of its existence. Next summer he was siezed with excruciating pain in his feet, which affected, successively, nearly all his joints (till it reached his head) in the form of a true inflammatory rheumatism, which after venesection, emetics, cathartics and digitatis required, as before, a resort to the bark.

It is well known that ophthalmia is an endemic of the Western country. Our earliest recollections connect themselves with this painful malady—peculiarly the disease of children of every age.

We have ourselves experienced it, more than in our boyhood, and remember to have seen whole families afflicted with it at the same time. It has always been worse in the country than in the towns, at the period, however, in which it prevailed most, the West had no considerable towns. Of its connection with autumnal fever, there can be no doubt. Twenty years ago, the writer of this article printed and distributed a little work, entitled '*Notices Concerning Cincinnati*,' in which the miasmatic origin of this malady was insisted upon, for reasons which are, perhaps, not unworthy of a more permanent record and a wider dissemination.

"OPHTHALMIA. On the arrangement of this affection among the miasmatic diseases, it is by no means intended to insist. The following are the reasons for which it was referred to that head, and physicians can estimate them, as they deserve. 1. The ophthalmia is an endemial disease of this country, which like our other endemics, appears sporadically every summer, and occasionally becomes epidemic, affecting great numbers, especially children 2. It occurs as much, (if not more,) along our water courses, and in the depths of forests, as on open plains or uplands ; and therefore neither dust, nor reflected light, has any agency in its production. 3. When epidemic, it appears and declines about the same time, with our other summer miasmatic diseases. 4. It has been prevalent before the annual burning of the woods, which invariably takes place in some parts of this country, and therefore is not occasioned by smoke. 5. In the summer of 1807, I was assured of two cases, in which this disease alternated with cholera infantum ; the ophthalmia prevailing at night, and the cholera infantum in the day. Similar cases have been mentioned to me by Dr. J. Canby. 6. This disease has diurnal exacerbations. It is generally worst at night, even where the eyes have not been exposed to the light. In one case, the subject of which (a man of veracity and observation) communicated the account to Dr. Este, of Hamilton, it assumed a tertian type. During the paroxysm, which had about the length of a common fit of fever and ague, light and every exertion of the eye were intolerable ; but during the intermission, he was entirely free from those morbid sensibilities. The same physician has also lately met with a case in his practice at that place, in which ophthalmia had true tertian paroxysms 7. Topical applications are seldom adequate to the cure, and means calculated to operate on the general system, must be resorted to in all violent cases. 8. It is somewhat difficult to conceive, how, either directly, or through the medium of the general system, the action of miasmata can be concentrated in the eye ; but there does not appear to be in it, any physical impossibility." 48.

We are enabled to state, that observations continued down to the present time, have but served to confirm the conclusions which our early experience suggested, and which at *that time* we supposed to be new. Within the last three years, a great number of patients afflicted with chronic ophthalmia, have applied at the Eye Infirmary ; and afforded, by the histories of their cases, abundant evidence, that this disease is, in general, dependent on the same cause with autumnal fever.

In the following paragraph, we entirely concert with our author.

"I formerly showed that the intermittent hemicrania sometimes affected one eye ; so as to produce lacrymation, and also a daily habitual inflammatory state of the eye. It is but to suppose this augmented and rendered more permanent, and we have the ophthalmia in question ; it is but to suppose the intermittent overlooked, as it most generally is in this state, and the ophthalmia will appear the sole disease, as every Neuralgia does to those who are equally inattentive. And that this is possible, is proved by the rheumatic or neuralgic inflammation of the face or jaws. The disease here may be mere pain, it may be pain with temporary or slender inflammation, or it may become permanent inflammation ; and moreover, it may also terminate in an abscess ; a fact, further, which occurs, if rarely, in the eye also. The analogy is perfect, as the cause is the same ; while we can find remoter, though not less instructive analogies, in the local and periodical rheumatisms of various parts, attending intermittent, where there is equally inflammation ; similarly variable in severity and duration, similarly periodical, and similarly transferrible, or interchangeable with other disorders of the same generic nature, or with those which attend anomalous intermittent." 365.

Passing from the anterior to the posterior part of the eye, Dr. M. enumerates amaurosis, among the consequences of the malady under consideration.

"To return. The other suggestions relates to Amaurosis ; and it rests similarly on some partial observations, in the first instance, but is better supported, both by analogy and an examination of recorded histories. The general analagy of all the Neuralgiæ tends to show, in the first place, that such is not an improbable event as the consequence of this disease, whether flowing immediately from improper treatment, or from their general tendency towards palsy. And if it is easy to understand how a paralysis of the retina might follow in any case where, in this ophthalmia, the neighbouring nerves had been affected, it is particularly obvious how it might be the termination of the simple Neuralgia of the optic nerve formerly described.

Again, to derive another analogy from intermittent fever, since the value of those has often been here demonstrated, I formerly noticed from authors, two cases of Amaurosis connected with that disorder ; suggesting then a possibility respecting its cause, which I must nevertheless, in some measure repeat here. They are, in any view, valuable as to the history of this hitherto almost incurable, and much neglected disease : and though to be ranked with the primary palsies arising from Malaria or intermittent, they will at least confirm the possibility of its also arising more indirectly, from the cause which I am here contemplating ; since this is, again, one of those involved cases of local and general action which are so difficult to separate, and which never cross me without causing me to regret that I have been here obliged to separate them." 370—371.

We have now under our care a countryman, living where ague and fever is annually epedemic, who was siezed last winter with periodical headach, in and about the right orbit, which speedily terminated (before we saw him) in perfect amaurosis of that eye, with dilated pupil. After blood letting ond cupping, he took the bark united with jal..p, with great advantage as it respects the neuralgia, and his pupil is nearly reduced to its natural size, but no return of vision has yet taken place.

We consider the Chapter now under review, as one of the most important in Dr. M.'s work ; but our limits do not admit of a further analysis.

Chapter XIX, contains a view of the connexion between neuralgia and intermittent. We shall only extract the summary of analogies and identities with which it closes.

"The abstract in question is therefore the following, passing over the community of remittent and intermittent fevers, as an admitted fact. Intermittent fevers arise from Malaria, certainly, as principally, and from mere cold possibly ; but are renewable by mere cold, when once they have existed.

They are often attended by peculiar local symptoms producing the anomalous varieties, while, when the febrile state is slight or obscure, these local disorders appear to be the chief disease.

Such local disorders are either affections of the nervous system, or of an inflammatory character, and they have been fully described.

The same intermittent fevers, more or less distinct, are accompanied by all the Neuralgiæ that have been described, whether these consist of simple pain, or are attended by inflammation ; and when the febrile state is slight or obscure, those local affections appear to form the chief disease.

If intermittent fevers alternate with all the anomalous local symptoms or diseases, so do they with all the neuralgic diseases : and in such cases, the supervention of one is the removal of the other.

Thus also, all those local diseases, including all the Neuralgiæ, alternate with each other ; or the appearance of one form is the cure of a preceding one.

Many of the Neuralgiæ will exist almost simultaneously, or else in alternating paroxysms ; these having any of the types of intermittent.

They also exist in alternating paroxysms with simple intermittent : or a particular doubled type will consist alternately of a paroxysm of pure fever and a paroxysm of Neuralgia.

The same individual under a persevering intermittent, will experience many of the anomalous forms of that disease, and also many of the neuralgic diseases, in alternation or succession ; or else in union ; and, in such cases, the type, and the hour of recurrence, will be the same for all the forms, even through a long course of years.

Malaria will produce the neuralgic diseases directly, as probably, will mere cold ; but they are renewable by mere cold when once they have existed ; and in these cases, though the intermittent fever is probably always present, it may be so slight as to be overlooked. In this, the first cause, Neuralgia, in all its forms, resembles intermittent : but it differs, inasmuch as it can be excited by direct injury of a nerve ; a difference, however, which is of no moment as to the general identity, because we know of no means of thus injuring the entire nervous system so as to produce general intermittent.

The same Malaria, in the same spot, acting on different individuals at the same time, will produce either intermittent or Neuralgia, and every form of each.

Intermittent and Neuralgia, in all their forms, are cured by the same remedies, and injured by the same wrong treatment ; and those remedies are constitutional ones, whether the diseases be local or general ; while, very particularly, the local and the general diseases both, are cured by operations on the imagination.

The conspicuously wrong treatment for all of these diseases, whether Neuralgiæ or intermittents, consists in the debilitating practice, as the right treatment is found in what is esteemed the reverse ; and whatever be the disease, be it local or general, when that practice is pushed so far as to become injurious, the injury is always of the same character, affecting the entire nervous system." 394—395.

The 20th Chapter is on 'Certain consequences of Intermittent or Marsh Fever and Neuralgia.' It contains nothing particularly new, or which is not a deduction from what has been already set forth, we shall, therefore, pass it over, and enter on the treatment of Neuralgia.

This is the subject of Chapter XXI, of which our limits will permit us to give but a brief abstract ; which, however, we regret the less, as the chapter is nearly a second edition of that on the cure of intermittent fever.

Like chronic intermittent, neuralgia often subsides spontaneously, and is, also, sometimes cured by charms, and other means which excite the imagination or feelings of the patient. It is to be regretted, however, that but little reliance can be placed on this *methodus medendi.* When the patient resides in an unhealthy situation, a removal should always be advised. Stimulants and narcotics, when the disease is regularly paroxysmal, if taken in anticipation of the fit, will frequently ward it off. But, in the opinion of our author, the latter class of medicines cannot be regarded as curative. In this decision, we entirely concur ; and would refer our readers to the history of an obstinate case of neuralgia, reported in the first number of this journal, in which nearly the whole catalogue of powerful narcotics were administered without effect, but rather to the injury of the patient.

According to our author, the true remedies for neuralgia are the tonics ; of which the bark and arsenic are the most important. In the treatment of intermittents, he gives a preference, as we have seen, to the former, but in neuralgia, he has found arsenic more efficacious. This, in fact, is his *great* remedy ; though in confirmed and protracted cases, he admits that it frequently fails. He thinks it superior to the carbonate of iron, which however, he would not wholly reject.

When tonics prove ineffectual, it is frequently owing to one of two causes,—first, the extreme irregularity of the paroxysms; and, secondly, the long continuance of the disease. Under these circumstances, he has known a single blood letting, an emetic, or a gentle salivation, especially when accompanied with a change of residence, so to act on the disease, or the constitution, as to render tonics effective in completing the cure.

In local applications, Dr. M. has very little confidence. He has seen but slight benefit from narcotics, thus applied. Of the salts of morphia he says nothing. It appears, however, that they have recently been found serviceable, when sprinkled on a blistered surface, after the removal of the cuticle. Publications to this effect have been made; and we have been informed, by our ingenious friend, Dr. La Roche, of the North American Journal, that in several late cases he has directed this practice, with advantage. Blisters, near the affected nerve, our author has, uniformly, found to aggravate the pain; but they have, occasionally, done good, when applied to a part somewhat remote. Nearly the same remark is applicable to issues and setons. Electricity and galvanism, he thinks, still less entitled to attention, having never seen them do the least good. The only topical application which he has known of advantage, is steam directed upon the affected part, through a tube,

Such is the catalogue of our authors remedies; which are followed by another of prohibited measures, a knowledge of which he thinks equally important. We shall briefly enumerate them. Excision of the affected nerve, he totally proscribes, as unphilosophical, unsuccessful, and cruel. Blood letting, cupping, and leeching he regards as generally useless, and carried to much extent, highly pernicious. Purging he views with equal disapprobation, and ridicules it as a 'fashionable folly.' Finally he eschews a low and bland diet, and advises the patient to live generously.

Such is the outline of Dr. M.'s *methodus medendi* in neuralgia. His confidence in its efficacy is great—rather too great, we apprehend. The well known influence of bark and arsenic in intermittent fever, taken in connexion with his *opinion*, that neuralgia is but a form of that disease in a chronic state, rather than practical experience, seem to us to be the foundation of this confidence. Certain it is, at all events, that both bark and arsenic have frequently failed to cure or even mitigate neuralgia. In the first number of this journal, already cited, we have published a case in which stimulants and tonics, both vegetable and mineral, were administered profusely without avail, after which the disease was signally mitigated, though not entirely removed by low diet, and the loss at different times by a hundred ounces of blood—most of which was sizy. We have, moreover, seen other cases, in which the same practice was beneficial—we cannot say absolutely curative. From such facts we are inclined to believe, that blood letting and other antiphlogistics are often necessary as preparatives in neuralgia, as we know they frequently are in intermittent fever. Sydenham long ago taught, that almost all diseases may be advantageously treated, in the first place, by bleeding and purging; and our own experience has convinced us, that these remedies, at the beginning of our curative efforts, are seldom injurious in *any* disease, while they very commonly give a beneficial effect to subsequent stimulation which it could not otherwise produce. It is an error to suppose that we must keep on with the method of treatment with which we set out. Stimulants and tonics are seldom proper as first remedies, in any form of disease, nor blood letting, cathartics and abstinence, as last. Depletives prepare the vital properties of the system, for being favourably acted on by excitants. It is loose phraseology to speak of these, as distinct and incompatible plans of cure. They are but the elements of one plan, and in most diseases neither of them can be rejected without rendering the treatment imperfect. The skill of the practitioner will be found in their proper combination, not in the selection of either. As well might a general decide, *a priori*, that he would employ nothing but artillery in repelling one invasion, infantry another, and cavalry a third, when the union, or successive operation, of every variety of troops might become necessary. By an ignorance, or neglect of this therapeutic maxim, we have seen much injury done to the sick. It is a modification of *Brunonianism*. The physician seeks to determine whether the disease is 'inflammatory' or 'nervous'—'sthenic or asthenic' 'sanguineous' or 'dyspeptic'—and acts accordingly. The moment his 'mind is made up' he opens *one* of his lockers, instead of the entire magazine, and proceeds to execute the 'plan of cure which the 'nature of the disease requires.' Is it a phlegmasia ?—then for the lancet, leeches and gum water, for 30 days. Are the bowels and liver torpid ? Give the blue pill by night, and cathartics by day, and keep on till the stools have a 'pleasant smell'—if this should be slow in happening, do not be 'weary in well doing' but throw in heavier doses, and wonder with the patient how he could hold so much bad matter. Is the disease of an intermitting type? Abjure the whole catalogue of depletions and abstractions, and begin, continue, and end, with the bark and arsenic.—Is the fever typhus or bilious? why, typhus. Then give him stimulants, narcotics, alexipharmics, sudorifics, and tonics; 'the rule requires it:' if you don't keep his strength up, it will 'run down:' If any thing can cure him this plan will: it is the right one, and if you let him die it is your own fault.—But is not the case a bilious fever? Yes, for the patient vomited yellow bile. Well then, of course, give calomel; repeat it, reiterate the repetition, and repeat the reiteration, till his liver is tired out; when, if he should not recover, you will have the satisfaction of knowing that you did your duty.

The last Chapter of Dr. M.'s work, is on the state of the nerves in neuralgia and intermittent. He wishes that this chapter should be considered in the light of an appendix of speculations to the essay, in which he was guided by 'evidence, experience and induction.' He believes, that in the diseases which are the subject of that essay, the nerves are *primarily* affected. What this affection is, he does not pretend to teach or understand. It is a departure from that condition, which enables them to perform their proper office in the animal economy, and it is the first effect of the remote cause. But our means of observation do not enable us to appreciate it anatomically. The congestions and other alterations of structure, which imply disruptions of the circulation, or disordered conditions of the chemistry of life, he regards as consequences of the primary læsion of the nervous function.

In intermittent fever there is, he thinks, an unhealthy state of the whole nervous system; in neuralgia there is a superadded morbid state of a particular nerve, generally induced by the former, or by its remote cause, but some times by other causes acting topically. The inflammations (so called) which, occasionally, show themselves in connexion with neuralgia, he views as distinct from true inflammation, the remedies for which, generally aggravate and can never cure them.

Back-wood's-men, as we are, and but little qualified for criticisms on the style and manner of the great writers of the old world, we must be permitted to say, that we have seldom met with a work more exceptionable in these respects than that which we have just travelled through. We do verily believe, that all which it contains might have been told in half the number of words. There is a playing round and never coming to the point, which we do not recollect ever to have seen equalled. Fvery thing seems preliminary with our author—you are not quite sure, that you are, at any time, in the middle of his argument or inquiry. His sentences are generally deficient in simplicity and too often in perspicuity ; many of them are parenthetical, and even whole paragraphs might advantageously be enclosed in ( ). The arrangement of his matter is so imperfect, that he felt it necessary, repeatedly, to assure his readers, that, in the present state of our knowledge of the matters which he treats, no better could be adapted,—an opinion from which we are compelled to dissent. We are, indeed, somewhat astonished, that one who could favour the profession with so great a mass of original observations and reflections, should not have been able to reduce them to a more natural method. He every where abounds in general reflections, most of which are excellent, *per se*, but often foreign to the subject which he is at the moment prosecuting, and calculated to divert the mind of the reader from it. Finally, his want of condensation shows itself in a great deficiency of propositions and corollaries, which distributed through a book of science, serve, in some degree, the purpose of the fixed stars in physical astronomy, by the aid of which the orbits and movements of the planets are calculated.

We are far, however, from wishing to detract from the merit or fame of Dr. M'Cullough, whose book, seems to us to be, in many respects, one of the most curious, original, and interesting, in its inquiries and results, that has lately fallen under our observation, while it every where abounds in evidences of zeal for the dignity of the profession, and breathes throughout a lofty spirit of candour and general benevolence.

